# Seroprevalence of SARS-CoV-2 Antibodies in Africa: A Systematic Review and Meta-Analysis

**DOI:** 10.3390/ijerph19127257

**Published:** 2022-06-14

**Authors:** Khalid Hajissa, Md Asiful Islam, Siti Asma Hassan, Abdul Rahman Zaidah, Nabilah Ismail, Zeehaida Mohamed

**Affiliations:** 1 Department of Medical Microbiology and Parasitology, School of Medical Sciences, Universiti Sains Malaysia, Kubang Kerian 16150, Malaysia; khalid541983@yahoo.com (K.H.); sitiasmakb@usm.my (S.A.H.); drzaidah@usm.my (A.R.Z.); drnabilah@usm.my (N.I.); 2 Department of Zoology, Faculty of Science and Technology, Omdurman Islamic University, P.O. Box 382, Omdurman 14415, Sudan; 3 Department of Haematology, School of Medical Sciences, Universiti Sains Malaysia, Kubang Kerian 16150, Malaysia; 4 Institute of Metabolism and Systems Research, University of Birmingham, Birmingham B15 2TT, UK; 5 Hospital Universiti Sains Malaysia, Universiti Sains Malaysia, Kubang Kerian 16150, Malaysia

**Keywords:** seroprevalence, SARS-CoV-2, COVID-19, antibodies, Africa, IgG, IgM, meta-analysis

## Abstract

A reliable estimate of SARS-CoV-2-specific antibodies is increasingly important to track the spread of infection and define the true burden of the ongoing COVID-19 pandemic. A systematic review and a meta-analysis were conducted with the objective of estimating the seroprevalence of SARS-CoV-2 infection in Africa. A systematic search of the PubMed, Scopus, Web of Science and Google Scholar electronic databases was conducted. Thirty-five eligible studies were included. Using meta-analysis of proportions, the overall seroprevalence of anti-SARS-CoV-2 antibodies was calculated as 16% (95% CI 13.1–18.9%). Based on antibody isotypes, 14.6% (95% CI 12.2–17.1%) and 11.5% (95% CI 8.7–14.2%) were seropositive for SARS-CoV-2 IgG and IgM, respectively, while 6.6% (95% CI 4.9–8.3%) were tested positive for both IgM and IgG. Healthcare workers (16.3%) had higher seroprevalence than the general population (11.7%), blood donors (7.5%) and pregnant women (5.7%). The finding of this systematic review and meta-analysis (SRMA) may not accurately reflect the true seroprevalence status of SARS-CoV-2 infection in Africa, hence, further seroprevalence studies across Africa are required to assess and monitor the growing COVID-19 burden.

## 1. Introduction

Coronavirus disease 2019 (COVID-19), a highly contagious disease caused by severe acute respiratory syndrome coronavirus 2 (SARS-CoV-2), continues to rapidly spread across the world. By 25 February 2022, more than 430 million COVID-19 cases had been confirmed and more than 5,922,049 COVID-19-related deaths had been documented globally [1]. The SARS-CoV-2 pandemic has placed an unprecedented pressure on healthcare systems across the world. Taking into account that no country was adequately prepared for such a quickly spreading pandemic, the consequences of this outbreak have challenged the sustainability of healthcare systems, even in developed countries [2]. In Africa, the pandemic has been projected to be devastating due to the continent’s poor health systems, gaps in medical infrastructure, and vulnerability to infectious diseases [3,4]. However, the COVID-19 infection rates in African countries are now significantly lower than in other continents.

According to the Africa CDC, a total of 11,129,366 confirmed cases, 247,310 deaths and 10,331,607 recoveries had been documented in Africa by 25 February 2022 [5]. Indeed, the current statistics on the number of confirmed cases and deaths are useful in tracking the dynamics of the disease transmission; however, they are insufficient for estimating the proportion of the infected population [6]. Until now, most African countries have had limited access to viral testing by RT-PCR to screen all SARS-CoV-2 suspected patients or those are at risk of infection due to infrastructure limitations and intermittent supply shortages. In general, mild or asymptomatic individuals are often not screened and thus, the reported cases are unlikely to reflect all SARS-CoV-2 infections [7,8]. Accordingly, the true magnitude of this outbreak is most likely underestimated. In this context, seroprevalence estimates using anti-SARS-CoV-2 antibodies as markers of viral exposure are of utmost importance to identify the proportion of the previously infected population [9]. Detecting anti-SARS-CoV-2 antibodies (IgM or/and IgG) may accurately capture the true cumulative prevalence of SARS-CoV-2 infection [10], which is essential for better understanding the course and extent of the ongoing COVID-19 pandemic [11], the contagiousness and the immunity against SARS-CoV-2 in vulnerable individuals as well as the community [12]. Furthermore, data on SARS-CoV-2 seroprevalence amongst African population is important for assessing the success of the current public health interventions.

Seroprevalence investigations have been undertaken on a worldwide scale to provide insight into SARS-CoV-2 epidemiology. Monitoring changes in seroprevalence data over time is essential for anticipating the dynamics of any pandemic and planning an effective public health response. Accordingly, few systematic reviews have comprehensively synthesised seroprevalence findings related to anti-SARS-CoV-2 antibodies amongst the general or targeted group of the population. However, with the significant expansion of relevant literature, having an updated picture of anti-SARS-CoV-2 seroprevalence is critical. With this background in mind, this SRMA was conducted to estimate the seroprevalence rate of SARS-CoV-2 in Africa.

## 2. Materials and Methods

### 2.1. Literature Search Strategy

In this study, a literature search, a study selection and reporting of the results were conducted on the basis of Preferred Reporting Items for Systematic Reviews and Meta-Analyses (PRISMA) (Table S1) [13]. The protocol of this SRMA was registered on the International Prospective Register of Systematic Reviews (PROSPERO) database (registration number: CRD42021250601). A total of 4 electronic databases, namely, PubMed, Scopus, Web of Science and Google Scholar were systematically searched for studies published up to 1 July 2021, and those reporting the data on the seroprevalence of SARS-CoV-2 infection amongst African population without language restriction. The detailed search strategy that was used for all databases is shown in Supplementary Table S2. In addition, reference lists of retrieved articles were tracked for identification of further relevant studies.

### 2.2. Data Management and Study Selection

At the initial stage, all of the identified records were combined in EndNote X9 (Clarivate Analytics, London, UK). A strategy involving both auto- and hand-search was used for identification and removal of duplicates before the titles and abstracts of the remaining records were independently assessed for inclusion by three reviewers (K.H., Z.A.R. and N.I.). Subsequently, the full texts of the potentially eligible records were obtained and assessed for eligibility by two reviewers (S.A.H. and Z.M.). Any discrepancies or uncertainties were resolved by discussion and consensus.

### 2.3. Eligibility Criteria

The outcome of interest in this study is the seroprevalence of SARS-CoV-2 infections in Africa. Overall seroprevalence was defined as detection of SARS-CoV-2-specific IgG and IgM in combination or separately. Accordingly, original studies from African countries that report information on the seroprevalence of SARS-CoV-2 antibodies were considered eligible for inclusion, whilst comments, case reports, editorials and reviews were excluded. In addition, studies of non-human subjects or non-serological investigations were excluded.

### 2.4. Quality Assessment

Two reviewers (K.H. and M.A.I.) independently used the critical appraisal tool developed in the Joana Brigg’s Institute (JBI) for prevalence studies [14] to assess the methodological quality of each included study. The assessment results were further validated by the other authors and notable discrepancies were identified resolved by verification and discussion. The tool contains nine items and each of them corresponds to a ‘yes’, ‘no’, ‘unclear’ or ‘not applicable’. Each study was assessed on the basis of the proportion of ‘yes’ answers given to the items. The high proportion (≥70%) of ‘yes’ refers to high-quality study (low risk of bias), whilst the moderate (50–69%) and low proportion (≤49%) of ‘yes’ refer to moderate- and low-quality studies, respectively [15,16,17].

### 2.5. Data Extraction

Following a full text review, relevant information was extracted by one reviewer (K.H.) using predesigned data collection sheet and later verified by four other reviewers (S.A.H., Z.A.R., N.I. and Z.M.). For all of the qualifying records, principal data were extracted on the number of subjects who were quantitatively or qualitatively tested for anti-SARS-CoV-2 antibody and how many were seropositive. In addition, information on the following variables were extracted: first author’s name, publication year, study design, country and place where the study was conducted, target population, recruitment location, gender, age, tested antibodies, serodiagnostic test and sensitivity and specificity of antibody tests. The United Nations Statistics Division African Region (Southern, Western, Central, Eastern and Northern Africa) was assigned to each study in accordance with the country of recruitment.

### 2.6. Statistical Analysis

Seroprevalence was calculated as the ratio of seropositive individuals to the total participants by using the Metaprop command. Accordingly, the seroprevalence of SARS-CoV-2 antibodies [at 95% confidence intervals (CI)] was estimated for each included study and subsequently for Africa by pooling the seroprevalence rates of all studies through the use of the random-effect model. Heterogeneity between the studies was evaluated using *I*^2^ statistics in conjunction with Cochran’s Q-test. A cut-off value ≥ 75% of *I*^2^ statistic was used to indicate substantial heterogeneity [18], whilst a *p* value of <0.05 was considered to be a significant degree of heterogeneity. Publication bias was examined graphically using a funnel plot and statistically by Egger’s regression test.

### 2.7. Subgroup and Sensitivity Analysis

The potential sources of heterogeneity were further explored by estimating the seroprevalence of SARS-CoV-2 based on different subgroups, including antibody isotypes, antibody tests, target population, study setting and African regions. Furthermore, sensitivity analysis was performed by excluding (i) small studies (n < 200), (ii) low-quality studies (high risk of bias) and (iii) outlier studies.

## 3. Results

### 3.1. Study Selection

**Figure 1:**
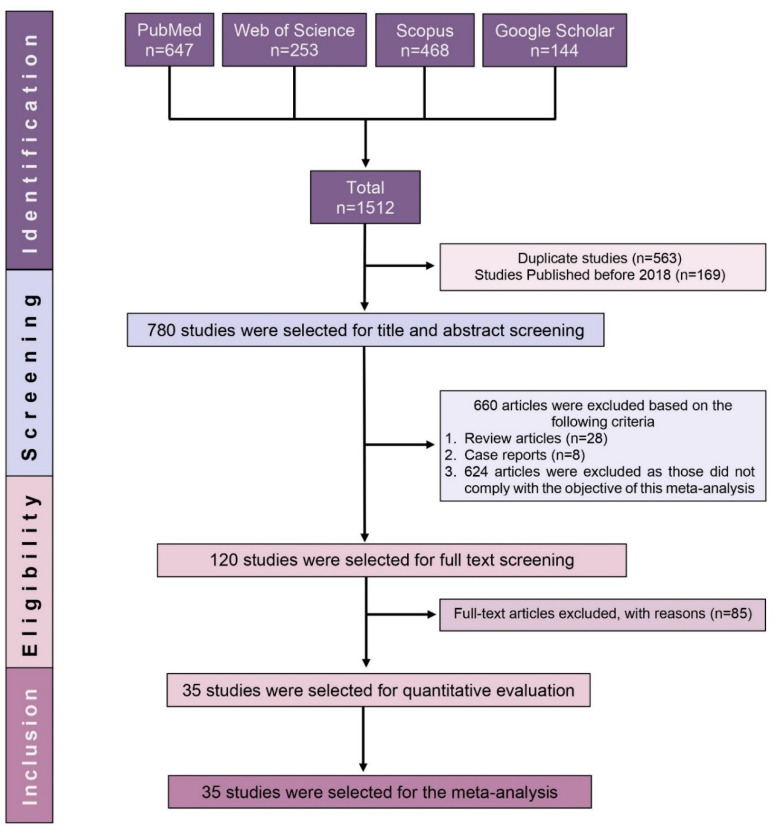
PRISMA flow diagram of study selection.

A total of 1512 records were retrieved in the initial search. Duplicates and studies published before 2018 were identified and removed, leaving 780 potential records. A further 660 studies were excluded following title and abstract screening. Subsequently, the full texts of the remaining 120 studies were assessed for eligibility, with 85 of them being excluded due to lack of seroprevalence data. Finally, only 35 fulfilled the criteria for inclusion in this SRMA (Figure 1).

### 3.2. Characteristics of Included Studies

Table 1 outlines the major characteristics of the 35 studies included in this SRMA. In total, 47,160 individuals recruited from 16 African countries and tested for the presence of anti-SARS-CoV-2 antibodies were included. Of the 35 eligible studies, 31 are published articles and 4 are preprints. A total of 10 studies were conducted in Ethiopia and Kenya (5 studies each); 4 studies were conducted in Egypt, 3 were conducted each in Democratic Republic of the Congo and South Africa; 2 studies were carried out each in Cameroon, Libya and Zambia and 1 study was conducted in each of the following countries: Angola, Ghana, Ivory Coast, Malawi, Nigeria, South Sudan, Republic of the Congo, Togo and Zimbabwe. The regional distribution of the included studies revealed that 15 studies were from Eastern Africa, 7 studies were from Central Africa, 3 studies were from Southern Africa and 6 were each from Northern and Western Africa. The included studies utilised various serodiagnostic assays. The numbers of studies that used rapid diagnostic test (RDTs), ELISA, chemiluminescent microparticle immunoassay (CMIA), chemiluminescence immunoassay (CLIA) and other tests were 15, 10, 3, 2 and 3, respectively.

### 3.3. Seroprevalence of SARS-CoV-2 Antibodies

**Figure 2:**
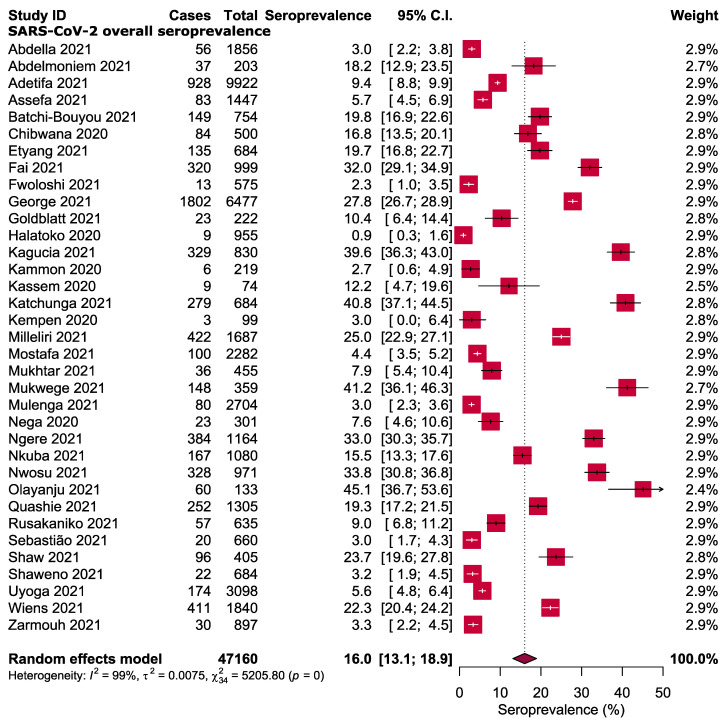
Overall seroprevalence of SARS-CoV-2 antibodies in Africa [19,20,21,22,23,24,25,26,27,28,29,30,31,32,33,34,35,36,37,38,39,40,41,42,43,44,45,46,47,48,49,50,51,52,53].

The forest plot in Figure 2 shows the estimated seroprevalence of anti-SARS-CoV-2 antibodies from the included studies and the corresponding 95% CI. The lowest rate of anti-SARS-CoV-2 antibodies was 0.9% in Togo [30], whilst the highest of 45.1% was reported in Nigeria [45]. By using the random-effect model, the pooled overall seroprevalence of anti-SARS-CoV-2 antibodies was calculated as 16.0%, with a heterogeneity of *I*^2^ 99% (*p* < 0.001, Figure 2).

### 3.4. Subgroup Analysis

**Figure 3:**
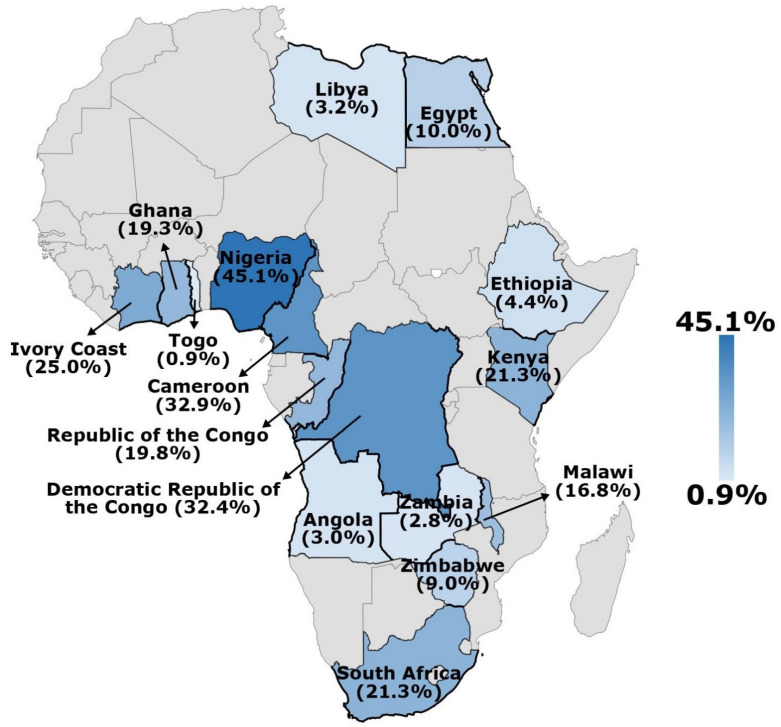
Overall seroprevalence of SARS-CoV-2 antibodies in different countries in Africa.

Subgroup analysis was carried out to identify factors that may have contributed to the high degree of heterogeneity. Table 2 and Figure S1 show the seroprevalence of SARS-CoV-2 across various subgroups. The pooled seroprevalence of SARS-CoV-2 infection based on antibody isotypes showed that 14.6% participants reported in 30 studies were seropositive for IgG against SARS-CoV-2, 11.5% recruited in 15 studies were seropositive for IgM and 6.6% of them in nine studies were tested positive for IgM and IgG. Some similar overall estimates were observed when anti-SARS-CoV-2 antibodies were measured by ELISA (16.5%), CMIA (9.8%) and RDTs (15.5%), whilst it was slightly low when CLIA was used (15.6%). Furthermore, the overall seroprevalence of SARS-CoV-2 antibodies in different countries in Africa is presented in Figure 3.

The pooled estimates of SARS-CoV-2 seroprevalence was highest amongst the participants recruited from hospitals (21.9%), followed by community (16.7%), healthcare facilities (10.6%) and blood transfusion centres (7.5%). Based on the target group, HCWs were found to have a seroprevalence of 16.3%; the general population had 11.7% and blood donors and pregnant women had 7.5% and 5.7%, respectively. The seroprevalence of anti-SARS-CoV-2 antibodies based on the geographical regions of Africa was 26.5% in Central Africa, 22.0% in Western Africa, 20.7% in Southern Africa, 12.1% in Eastern Africa and only 6.5% in Northern Africa.

### 3.5. Quality Assessment and Publication Bias

Detailed information about the quality assessment of all included studies is presented in Supplementary Table S3. According to the JBI rating system, the risk of bias was high in 5 (14.3%) studies, moderate in 12 (34.3%) studies and low in 18 (51.4%) studies. As visually illustrated by the asymmetrical funnel plot and statistically confirmed by Egger test (*p* = 0.002), an evidence of significant publication bias was found within the included studies (Figure S2).

### 3.6. Sensitivity Analysis

Sensitivity analysis revealed that excluding small studies, low-quality studies and outlier studies (Figure S3) had no significant effect on the overall seroprevalence of anti-SARS-CoV-2 antibodies. The seropositivity rate remained within the 95% CI of the respective overall seroprevalence (Figure S4), indicating that the results generated in this SRMA are robust and reliable.

## 4. Discussion

The current uncertainties around the real counts of SARS-CoV-2 cases in Africa highlighted the need for obtaining credible seroprevalence estimates of SARS-CoV-2 antibodies, which may indicate the true extent of the COVID-19 pandemic in the region. Indeed, several previous investigations have verified that the actual number of infected individuals are much higher than the reported cases [54,55]. In this SRMA, the relevant literature was critically reviewed to provide an updated overview of the continent-wide SARS-CoV-2 seroprevalence.

The meta-analysis of data obtained from 47,160 individuals in 35 eligible studies showed a considerable variation in the seroprevalence of SARS-CoV-2 antibodies amongst the included studies and thus between African nations. The results revealed that the SARS-CoV-2 seropositivity amongst African population ranged from 0.9% to 45.1%, with an estimated overall seroprevalence of 16.0%. This finding is higher than the previous estimates of global seroprevalence [56,57]. It is also higher than the results of other population-based studies and nationwide serosurvey conducted in Europe [58,59,60], USA [61,62] India (7.1%) [63] and Brazil [64]. By contrast, it is relatively low compared with other rates reported from Iran, USA, Sweden, and India [54,65,66,67]. The observed variations in seroprevalence estimates between studies, countries and regions may be attributed to a number of factors, including public health responses, adherence to control measures and differences in community transmission. In addition, variations in seropositivity reflect the differences in study designs, study populations, antibody tests and data collecting dates.

Indeed, additional information is required before using serological testing as a sole basis to confirm or exclude active SARS-CoV-2 infection [68]. Studies are urgently needed to assess the kinetics of the SARS-CoV-2 antibody response and how it could be used to interpret serological results. Until then, the detection of SARS-CoV-2-specific IgG and IgM antibodies in combination with molecular testing could significantly improve COVID-19 diagnosis and play an essential role in assessing immunological responses following SARS-CoV-2 infection [69]. In the present SRMA, the pooled seroprevalence of IgG, IgM, and IgG and IgM antibodies against SARS-CoV-2 were estimated to be 14.6%, 11.5%, and 6.6%, respectively. However, the relatively high rate of positive IgG cases in this study was lower than the 67.44% found in a previous meta-analysis [70]. Similar to numerous other systematic reviews, various serodiagnostic platforms are available for the detection of SARS-CoV-2 antibodies, with RDTs being the most commonly used. In fact, comparing seroprevalence rates across studies is confounded by the variable efficiency of diagnostic tests. However, slight variations in the overall estimates were found when anti-SARS-CoV-2 antibodies were assessed using ELISA, CMIA and RDTs, possibly because the majority of diagnostic tests used have high sensitivity and specificity.

Subgroup analysis regarding the serological status of SARS-CoV-2 according to study population and recruitment site was conducted. As expected, the highest burden of SARS-CoV-2 antibodies were found amongst HCWs (16.3%) compared with general population (11.7%), blood donors (7.5%) and pregnant women (5.7%). Several studies have highlighted the potential of SARS-CoV-2 occupational transmission amongst HCWs, as they are on the frontlines of the COVID-19 response and, in turn, are more vulnerable to viral transmission [71,72,73]. The seropositivity rate amongst African HCWs was higher than the pooled estimates of many previous meta-analysis studies that included a global or regional data representation [74,75,76]. However, it is unknown whether HCWs have a higher seroprevalence rate as a result of their vaccination status, considering the fact that HCWs prioritised COVID-19 vaccination. Although little is known regarding the extent to which African HCWs adhere to infection prevention and control measures, compliance with to these mitigation measures and the appropriate use of personal protective equipment are critical for reducing infection risk amongst HCWs [77,78].

On the basis of the geographical regions of Africa, the meta-analysis results indicated that Central and Western Africa had higher seroprevalence rates (26.5% and 22.0%, respectively) than Southern (20.7%), Eastern (12.1%) and Northern (6.5%) Africa. Considering the limited evidence regarding the influence of environmental and demographic factors on SARS-CoV-2 transmission [79], neither the high seropositivity in Central Africa nor the low seropositivity in Northern Africa could accurately reflect the real status in these regions due to the few number of eligible studies in some regions. For instance, only one nation from Southern Africa and two from Northern Africa were included. Indeed, finding of one or two studies is inconclusive and should not be generalised. Since data from only 16 African countries were included in the analysis, a considerable proportion of SARS-CoV-2 infections in Africa remain unreported. The lack of baseline data on the seroprevalence of anti-SARS-CoV-2 in many African countries is critical because current information is necessary to understand the spread of COVID-19 in those countries. Therefore, further surveillance of SARS-CoV-2 seroprevalence is required to assess and monitor the increasing COVID-19 burden.

As this SRMA provides the most up-to-date comprehensive estimation of SARS-CoV-2 seroprevalence on the basis of a critical review of available literature, its findings should be interpreted in the context of several important limitations. Firstly, only 16 of the 54 African countries were included in this study. Besides, adequate representation in studies conducted in Southern and Northern Africa is lacking. Secondly, the reported seroprevalence in individual studies may be underestimated or overestimated depending on the sensitivity and specificity of the antibody test used. Furthermore, some of the included studies did not report the sensitivity and specificity of the antibody tests. Thirdly, a high level of heterogeneity was detected between individual studies and the asymmetry of the funnel plot suggested the existence of publication bias, both of which are common in such meta-analysis.

## 5. Conclusions

In conclusion, based on the comprehensive review and meta-analysis of available data on SARS-CoV-2 until 1 July 2021, the seroprevalence of anti-SARS-CoV-2 in Africa was estimated to be 16.0%. However, due to the limited number of relevant studies, the high level of heterogeneity and the large gap in studies conducted in most African countries, the findings of this SRMA may not accurately reflect the true seroprevalence status of SARS-CoV-2 infection in Africa. Therefore, further seroprevalence studies across Africa are required to assess and monitor the growing COVID-19 burden. However, in order to better interpret the results and provide clinically meaningful and valuable recommendations to healthcare providers and test recipients, several factors must be considered when conducting such seroprevalence studies: whether the person is symptomatic or asymptomatic at the time of testing; positive or negative status of IgM and IgG; as well as the test device quality.

## Figures and Tables

**Table 1:** Major characteristics of the included studies.

Study ID [References]	Country	Study Period	Sample Size	Target Population	Recruitment Location	Method	Investigated Antibodies
Abdella 2021 [19]	Ethiopia	July to September 2020	1856	General population	Community based	RDT	IgM/IgG
Abdelmoniem 2021 [20]	Egypt	1 to 14 June 2020	203	HCWs	Hospital	RDT	IgM and IgG
Adetifa 2021 [21]	Kenya	April to September 2020	9922	Blood donors	Blood transfusion centre	ELISA	IgG
Assefa 2021 [22]	Ethiopia	March to April 2021	1447	Pregnant women	Health facilities	RDT	Total antibodies
Batchi-Bouyou 2021 [23]	The Republic of Congo	April to July 2020	754	General population	Community based	RDT	IgM and IgG
Chibwana 2020 [24]	Malawi	22 May to 19 June 2020	500	HCWs	Hospital	ELISA	IgG
Etyang 2021 [25]	Kenya	30 July to 4 December 2020	684	HCWs	Hospital	ELISA	IgG
Fai 2021 [26]	Cameroon	June to August 2020	999	Symptomatic and Asymptomatic	Community and Hospital	RDT	IgM and IgG
Fwoloshi 2021 [27]	Zambia	July 2020	575	HCWs	Health facilities	ELISA	IgG
George 2021 [28]	South Africa	August to October 2020	6477	Outpatient	Hospital	CLIA	IgM and IgG
Goldblatt 2021 [29]	South Africa	1 May to mid-July 2020	222	HCWs	Hospital	ELISA	IgG
Halatoko 2020 [30]	Togo	April to May 2020	955	Non-Specific	Multiple settings	RDT	IgM and IgG
Kagucia 2021 [31]	Kenya	September to October 2020	830	Others	Community based	ELISA	IgG
Kammon 2020 [32]	Libya	April to May 2020	219	General population	Community and Hospital	RDT	IgM/IgG
Kassem 2020 [33]	Egypt	1 to 14 June 2020	74	HCWs	Hospital	RDT	IgM and IgG
Katchunga 2021 [34]	DRC	May to August 2020	684	Others	Health facilities	RDT	IgM and IgG
Kempen 2020 [35]	Ethiopia	May 2020	99	General population	Health facilities	CMIA	IgG
Milleliri 2021 [36]	Ivory Coast	July to October 2020	1687	Others	Community based	RDT	IgG/IgM
Mostafa 2021 [37]	Egypt	April to June 2020	2282	HCWs	Health facilities	RDT	IgM and IgG
Mukhtar 2021 [38]	Egypt	May to June 2020	455	HCWs	Hospital	RDT and CLIA	IgG
Mukwege 2021 [39]	DRC	July to August 2020	359	HCWs	Hospital	RDT and ELISA	IgM and IgG
Mulenga 2021 [40]	Zambia	4 to 27 July 2020	2704	General population	Community based	ELISA	IgG
Nega 2020 [41]	Ethiopia	23 to 28 April 2020	301	General population	Community based	RDT	IgG/IgM
Ngere 2021 [42]	Kenya	November 2020	1164	General population	Community based	EIA	IgM and IgG
Nkuba 2021 [43]	DRC	October to November 2020	1080	General population	Community based	Luminex-based assay	IgG
Nwosu 2021 [44]	Cameroon	October to November 2020	971	General population	Community based	RDT	IgM/IgG
Olayanju 2021 [45]	Nigeria	December 2019 to April 2020	133	HCWs	Hospital	ELISA	IgG
Quashie 2021 [46]	Ghana	July to September 2020	1305	Non-Specific	Multiple settings	RDT	IgM and IgG
Rusakaniko 2021 [47]	Zimbabwe	June 2020	635	HCWs	Health facilities	RDT	IgG and IgM
Sebastião 2021 [48]	Angola	July to September 2020	660	General population	community based	ELFA	IgM and IgG
Shaw 2021 [49]	South Africa	17 August to 4 September	405	Others	Community based	CMIA	IgG
Shaweno 2021 [50]	Ethiopia	June to July 2020	684	General population	Community based	CMIA	IgG
Uyoga 2021 [51]	Kenya	April to June 2020	3098	Blood donors	Blood transfusion centre	ELISA	IgG
Wiens 2021 [52]	South Sudan	August to September 2020	1840	General population	Community based	ELISA	IgG
Zarmouh 2021 [53]	Libya	18 to 21 April 2020	897	General population	Community based	CLIA	IgM and IgG

Key: DRC: Democratic Republic of the Congo, HCWs: Healthcare workers, RDT: Rapid Diagnostic Test, ELISA: Enzyme-linked Immunosorbent Assay, CLIA: Chemiluminescence Immunoassay, CMIA: Chemiluminescent Microparticle Immunoassay, EIA: Enzyme immunoassay and ELFA: Enzyme-linked fluorescent assay.

**Table 2:** Subgroup analyses estimating the pooled seroprevalence of SARS-CoV-2 antibodies in Africa.

Subgroups	Pooled Seroprevalence [95% CIs] (%)	Number of Studies Analysed	Total Number of Patients
**Antibody isotypes**			
Overall IgG	14.6 [12.2–17.1]	30	34,113
Overall IgM	11.5 [8.7–14.2]	15	10,882
IgG and IgM	6.6 [4.9–8.3]	9	7557
**Antibody tests**			
Rapid diagnostic test	15.5 [11.0–20.1]	15	14,372
Enzyme-linked immunosorbent assay	16.5 [12.2–20.8]	10	20,508
Chemiluminescent microparticle immunoassay	9.8 [0.0–20.7]	3	1188
Chemiluminescence Immunoassay	15.6 [0.0–39.6]	2	7347
**Target population**			
General population	11.7 [7.4–16.0]	13	13,229
Healthcare workers	16.3 [11.5–21.2]	11	6122
Blood donors	7.5 [3.8–11.2]	2	13,020
Pregnant women	5.7 [4.5–6.9]	1	1447
**Settings**			
Community	16.7 [11.7–21.8]	14	15,833
Hospital	21.9 [14.8–29.0]	9	9107
Healthcare facilities	10.6 [5.1–16.1]	6	5722
Blood transfusion centre	7.5 [3.8–11.2]	2	13,020
**Regions**			
Northern Africa	6.5 [4.1–8.9]	6	4130
Central Africa	26.5 [14.6–38.4]	7	5507
Eastern Africa	12.1 [8.8–15.3]	15	26,339
Western Africa	22.0 [6.7–37.6]	4	4080
Southern Africa	20.7 [10.4–31.1]	3	7104

CIs: confidence intervals.

## Data Availability

The data generated in this study are available within the manuscript and Supplementary Materials.

## Supplementary Materials

The following supporting information can be downloaded at: https://www.mdpi.com/article/10.3390/ijerph19127257/s1, Table S1: PRISMA checklist. Table S2: Detailed Search Strategy. Table S3: Quality assessment of the included studies. Figure S1: Subgroup analyses. Figure S2: Funnel plot. Figure S3: Galbraith plot. Figure S4: Sensitivity analysis.
