# Supplementary material for: Seroprevalence of SARS-CoV-2 Antibodies in Africa: A Systematic Review and Meta-Analysis
Source: Int J Environ Res Public Health. 2022 Jun 14;19(12):7257. doi: 10.3390/ijerph19127257 (PMC9223681; doi:10.3390/ijerph19127257)
Supplement: Supplementary file 1 [file ijerph-19-07257-s001.zip › ijerph-1708668-supplementary.pdf]

Seroprevalence of SARS-CoV-2 antibodies in Africa: A systematic review and meta-analysis

Table S1: PRISMA 2020 Checklist

Section and Topic	Item #	Checklist item	Location where item is reported
**TITLE**			
Title	1	Identify the report as a systematic review.	1
**ABSTRACT**			
Abstract	2	See the PRISMA 2020 for Abstracts checklist.	2
**INTRODUCTION**			
Rationale	3	Describe the rationale for the review in the context of existing knowledge.	3-4
Objectives	4	Provide an explicit statement of the objective(s) or question(s) the review addresses.	5
**METHODS**			
Eligibility criteria	5	Specify the inclusion and exclusion criteria for the review and how studies were grouped for the syntheses.	5-6
Information sources	6	Specify all databases, registers, websites, organisations, reference lists and other sources searched or consulted to identify studies. Specify the date when each source was last searched or consulted.	5
Search strategy	7	Present the full search strategies for all databases, registers and websites, including any filters and limits used.	Table S2
Selection process	8	Specify the methods used to decide whether a study met the inclusion criteria of the review, including how many reviewers screened each record and each report retrieved, whether they worked independently, and if applicable, details of automation tools used in the process.	5
Data collection process	9	Specify the methods used to collect data from reports, including how many reviewers collected data from each report, whether they worked independently, any processes for obtaining or confirming data from study investigators, and if applicable, details of automation tools used in the process.	6-7
Data items	10a	List and define all outcomes for which data were sought. Specify whether all results that were compatible with each outcome domain in each study were sought (e.g. for all measures, time points, analyses), and if not, the methods used to decide which results to collect.	6-7
	10b	List and define all other variables for which data were sought (e.g. participant and intervention characteristics, funding sources). Describe any assumptions made about any missing or unclear information.	6-7
Study risk of bias assessment	11	Specify the methods used to assess risk of bias in the included studies, including details of the tool(s) used, how many reviewers assessed each study and whether they worked independently, and if applicable, details of automation tools used in the process.	6
Effect measures	12	Specify for each outcome the effect measure(s) (e.g. risk ratio, mean difference) used in the synthesis or presentation of results.	7
Synthesis methods	13a	Describe the processes used to decide which studies were eligible for each synthesis (e.g. tabulating the study intervention characteristics and comparing against the planned groups for each synthesis (item #5)).	7
	13b	Describe any methods required to prepare the data for presentation or synthesis, such as handling of missing summary statistics, or data conversions.	7
	13c	Describe any methods used to tabulate or visually display results of individual studies and syntheses.	7
	13d	Describe any methods used to synthesize results and provide a rationale for the choice(s). If meta-analysis was performed, describe the model(s), method(s) to identify the presence and extent of statistical heterogeneity, and software package(s) used.	7
	13e	Describe any methods used to explore possible causes of heterogeneity among study results (e.g. subgroup analysis, meta-regression).	7

Section and Topic	Item #	Checklist item	Location where item is reported
	13f	Describe any sensitivity analyses conducted to assess robustness of the synthesized results.	
Reporting bias assessment	14	Describe any methods used to assess risk of bias due to missing results in a synthesis (arising from reporting biases).	7
Certainty assessment	15	Describe any methods used to assess certainty (or confidence) in the body of evidence for an outcome.	NA
**RESULTS**			
Study selection	16a	Describe the results of the search and selection process, from the number of records identified in the search to the number of studies included in the review, ideally using a flow diagram.	7-8
	16b	Cite studies that might appear to meet the inclusion criteria, but which were excluded, and explain why they were excluded.	NA
Study characteristics	17	Cite each included study and present its characteristics.	10-11
Risk of bias in studies	18	Present assessments of risk of bias for each included study.	Table S3
Results of individual studies	19	For all outcomes, present, for each study: (a) summary statistics for each group (where appropriate) and (b) an effect estimate and its precision (e.g. confidence/credible interval), ideally using structured tables or plots.	12, 14-16
Results of syntheses	20a	For each synthesis, briefly summarise the characteristics and risk of bias among contributing studies.	NA
	20b	Present results of all statistical syntheses conducted. If meta-analysis was done, present for each the summary estimate and its precision (e.g. confidence/credible interval) and measures of statistical heterogeneity. If comparing groups, describe the direction of the effect.	12, 14-16
	20c	Present results of all investigations of possible causes of heterogeneity among study results.	15-16
	20d	Present results of all sensitivity analyses conducted to assess the robustness of the synthesized results.	19
Reporting biases	21	Present assessments of risk of bias due to missing results (arising from reporting biases) for each synthesis assessed.	NA
Certainty of evidence	22	Present assessments of certainty (or confidence) in the body of evidence for each outcome assessed.	NA
**DISCUSSION**			
Discussion	23a	Provide a general interpretation of the results in the context of other evidence.	19-21
	23b	Discuss any limitations of the evidence included in the review.	21
	23c	Discuss any limitations of the review processes used.	NA
	23d	Discuss implications of the results for practice, policy, and future research.	32
**OTHER INFORMATION**			
Registration and protocol	24a	Provide registration information for the review, including register name and registration number, or state that the review was not registered.	5
	24b	Indicate where the review protocol can be accessed, or state that a protocol was not prepared.	5
	24c	Describe and explain any amendments to information provided at registration or in the protocol.	NA
Support	25	Describe sources of financial or non-financial support for the review, and the role of the funders or sponsors in the review.	NA
Competing interests	26	Declare any competing interests of review authors.	29
Availability of data, code and other materials	27	Report which of the following are publicly available and where they can be found: template data collection forms; data extracted from included studies; data used for all analyses; analytic code; any other materials used in the review.	30

From: Page MJ, McKenzie JE, Bossuyt PM, Boutron I, Hoffmann TC, Mulrow CD, et al. The PRISMA 2020 statement: an updated guideline for reporting systematic reviews. BMJ 2021;372:n71. doi: 10.1136/bmj.n71

For more information, visit: http://www.prisma-statement.org/

Table S2: **Detailed Search Strategy**

Databases	Search Strategy
**PubMed**	((((((((((((((Prevalence[Title/Abstract])) OR (Seroprevalence[Title/Abstract])) OR (Seroepidemiology[Title/Abstract])) OR (Proportion[Title/Abstract])) OR (Serosurvey[Title/Abstract])) OR (surveillance[Title/Abstract])) OR (Anti[Title/Abstract])) OR (Antibody[Title/Abstract])) OR (antibodies[Title/Abstract])) OR (IgG[Title/Abstract])) OR (IgM[Title/Abstract])) OR (IgA[Title/Abstract])) AND (((((((SARS[Title/Abstract]) OR (SARS-CoV-2[Title/Abstract])) OR (SARS-CoV2[Title/Abstract])) OR (COVID-19[Title/Abstract])) OR (COVID19[Title/Abstract])) OR (coronavirus[Title/Abstract])) OR (nCoV[Title/Abstract]))) AND ((Algeria[Title/Abstract]) OR (Egypt[Title/Abstract])) OR (Libya[Title/Abstract])) OR (Morocco[Title/Abstract])) OR ("South Sudan"[Title/Abstract])) OR (Sudan[Title/Abstract])) OR (Tunisia[Title/Abstract])) OR (Burundi[Title/Abstract])) OR (Comoros[Title/Abstract])) OR (Djibouti[Title/Abstract])) OR (Eritrea[Title/Abstract])) OR (Ethiopia[Title/Abstract])) OR (Kenya[Title/Abstract])) OR (Madagascar[Title/Abstract])) OR (Malawi[Title/Abstract])) OR (Mauritius[Title/Abstract])) OR (Mozambique[Title/Abstract])) OR (Rwanda[Title/Abstract])) OR (Seychelles[Title/Abstract])) OR (Somalia[Title/Abstract])) OR (Tanzania[Title/Abstract])) OR (Uganda[Title/Abstract])) OR (Zambia[Title/Abstract])) OR (Zimbabwe[Title/Abstract])) OR (Benin[Title/Abstract])) OR ("Burkina Faso"[Title/Abstract])) OR ("Cape Verde"[Title/Abstract])) OR ("Cote d'Ivoire"[Title/Abstract])) OR ("Ivory Coast"[Title/Abstract])) OR (Gambia[Title/Abstract])) OR (Ghana[Title/Abstract])) OR (Guinea[Title/Abstract])) OR (Guinea-Bissau[Title/Abstract])) OR (Liberia[Title/Abstract])) OR (Mali[Title/Abstract])) OR (Mauritania[Title/Abstract])) OR (Niger[Title/Abstract])) OR (Nigeria[Title/Abstract])) OR (Senegal[Title/Abstract])) OR ("Sierra Leone"[Title/Abstract])) OR (Togo[Title/Abstract])) OR (Angola[Title/Abstract])) OR (Cameroon[Title/Abstract])) OR ("Central African Republic"[Title/Abstract])) OR (Chad[Title/Abstract])) OR (Congo[Title/Abstract])) OR ("Equatorial Guinea"[Title/Abstract])) OR (Gabon[Title/Abstract])) OR ("Sao Tome"[Title/Abstract])) OR (Botswana[Title/Abstract])) OR (Lesotho[Title/Abstract])) OR (Namibia[Title/Abstract])) OR ("South Africa"[Title/Abstract])) OR (Swaziland[Title/Abstract]))
**Scopus**	TITLE-ABS (prevalence OR seroprevalence OR "Seroepidemiology" OR proportion OR serosurvey OR surveillance OR anti OR antibody OR antibodies OR igg OR igm OR iga) AND TITLE-ABS (sars OR sars-cov-2 OR sars-cov2 OR covid-19 OR covid19 OR coronavirus OR ncov) AND TITLE-ABS (Algeria OR Egypt OR Libya OR morocco OR "South Sudan" OR sudan OR Tunisia OR Burundi OR comoros OR Djibouti OR Eritrea OR Ethiopia OR Kenya OR Madagascar OR Malawi OR Mauritius OR Mozambique OR Rwanda OR Seychelles OR Somalia OR Tanzania OR Uganda OR Zambia OR Zimbabwe OR benin OR "Burkina Faso" OR "Cape Verde" OR "Cote d'Ivoire" OR "Ivory Coast" OR gambia OR Ghana OR guinea OR guinea-bissau OR Liberia OR mali OR Mauritania OR niger OR Nigeria OR Senegal OR "Sierra Leone" OR togo OR angola OR Cameroon OR "Central African Republic" OR chad OR congo OR "Equatorial Guinea" OR gabon OR "Sao Tome" OR Botswana OR Lesotho OR Namibia OR "South Africa" OR Swaziland)
**Web of**	TI/AB=(SARS OR COVID OR coronavirus OR nCoV) AND TI/AB=(Prevalence OR Seroprevalence OR Seroepidemiology OR Surveillance OR anti OR Antibody OR antibodies OR IgG OR IgM OR IgA) AND

**Science**	TI/AB=(Algeria OR Egypt OR Libya OR Morocco OR “South Sudan” OR Sudan OR Tunisia OR Burundi OR Comoros OR Djibouti OR Eritrea OR Ethiopia OR Kenya OR Madagascar OR Malawi OR Mauritius OR Mozambique OR Rwanda OR Seychelles OR Somalia OR Tanzania OR Uganda OR Zambia OR Zimbabwe OR Benin OR “Burkina Faso” OR “Cape Verde” OR “Cote d'Ivoire” OR “Ivory Coast” OR Gambia OR Ghana OR Guinea OR Guinea-Bissau OR Liberia OR Mali OR Mauritania OR Niger OR Nigeria OR Senegal OR “Sierra Leone” OR Togo OR Angola OR Cameroon OR “Central African Republic” OR Chad OR Congo OR “Equatorial Guinea” OR Gabon OR “Sao Tome” OR Botswana OR Lesotho OR Namibia OR “South Africa” OR Swaziland)
**Google Scholar**	SARS OR COVID OR coronavirus OR nCoV) (Prevalence OR Seroprevalence OR Seroepidemiology OR Surveillance OR anti OR Antibody OR antibodies OR IgG OR IgM OR IgA) (Algeria OR Egypt OR Libya OR Morocco OR “South Sudan” OR Sudan OR Tunisia OR Burundi OR Comoros OR Djibouti OR Eritrea OR Ethiopia OR Kenya OR Madagascar OR Malawi OR Mauritius OR Mozambique OR Rwanda OR Seychelles OR Somalia OR Tanzania OR Uganda OR Zambia OR Zimbabwe OR Benin OR “Burkina Faso” OR “Cape Verde” OR “Cote d'Ivoire” OR “Ivory Coast” OR Gambia OR Ghana OR Guinea OR Guinea-Bissau OR Liberia OR Mali OR Mauritania OR Niger OR Nigeria OR Senegal OR “Sierra Leone” OR Togo OR Angola OR Cameroon OR “Central African Republic” OR Chad OR Congo OR “Equatorial Guinea” OR Gabon OR “Sao Tome” OR Botswana OR Lesotho OR Namibia OR “South Africa” OR Swaziland)

Table S3: Quality assessment of the included studies.

No.	Study ID	Questions assessing included studies									Yes (%)
		**1**	**2**	**3**	**4**	**5**	**6**	**7**	**8**	**9**	
1	Abdella 2021	Y	Y	Y	Y	N	Y	Y	N	Y	77.8
2	Abdelmoniem 2021	Y	Y	Y	Y	Y	Y	Y	Y	Y	100
3	Adetifa 2021	Y	Y	Y	Y	N	Y	U	Y	Y	77.8
4	Assefa 2021	Y	Y	Y	Y	N	Y	U	N	Y	66.7
5	Batchi-Bouyou 2021	Y	U	N	Y	Y	Y	Y	Y	Y	77.8
6	Chibwana 2020	Y	Y	Y	Y	N	Y	Y	N	U	66.7
7	Etyang 2021	Y	Y	Y	Y	N	Y	Y	Y	Y	88.9
8	Fai 2021	U	Y	N	Y	Y	Y	Y	Y	U	66.7
9	Fwoloshi 2021	Y	Y	U	Y	N	Y	Y	Y	U	66.7
10	George 2021	Y	Y	Y	N	Y	Y	N	N	Y	66.7
11	Goldblatt 2021	Y	Y	U	N	N	Y	Y	Y	U	55.6
12	Halatoko 2020	Y	Y	Y	Y	Y	Y	Y	Y	Y	100
13	Kagucia 2021	Y	Y	U	Y	N	Y	Y	Y	U	66.7
14	Kammon 2020	Y	U	N	Y	Y	Y	N	N	U	44.5
15	Kassem 2020	Y	U	Y	Y	Y	Y	Y	Y	Y	88.9
16	Katchunga 2021	Y	U	N	Y	Y	Y	Y	Y	U	66.7
17	Kempen 2020	Y	N	N	N	N	Y	Y	Y	U	44.5
18	Milleliri 2021	Y	U	Y	Y	N	Y	Y	Y	U	66.7
19	Mostafa 2021	Y	U	Y	N	Y	Y	Y	Y	U	66.7
20	Mukhtar 2021	Y	Y	Y	Y	N	Y	Y	Y	Y	88.9
21	Mukwege 2021	Y	Y	Y	Y	N	Y	Y	Y	Y	88.9
22	Mulenga 2021	Y	Y	Y	Y	N	Y	Y	Y	Y	88.9
23	Nega 2020	Y	U	N	Y	N	Y	Y	N	N	44.5
24	Ngere 2021	Y	Y	Y	Y	N	Y	Y	N	Y	77.8
25	Nkuba 2021	Y	Y	Y	Y	N	Y	Y	Y	Y	88.9
26	Nwosu 2021	Y	Y	Y	Y	Y	Y	Y	Y	Y	100
27	Olayanju 2021	Y	U	N	N	N	Y	Y	Y	U	44.5
28	Quashie 2021	Y	U	U	N	N	Y	Y	Y	U	44.5
29	Rusakaniko 2021	Y	Y	N	Y	Y	Y	Y	Y	U	77.8
30	Sebastião 2021	Y	N	Y	Y	Y	Y	Y	Y	U	77.8
31	Shaw 2021	Y	U	N	Y	N	Y	Y	Y	U	55.6
32	Shaweno 2021	Y	Y	N	Y	N	Y	Y	Y	U	66.7
33	Uyoga 2021	Y	U	Y	Y	N	Y	Y	Y	Y	77.8
34	Wiens 2021	Y	Y	Y	Y	N	Y	Y	Y	Y	88.9
35	Zarmouh 2021	Y	Y	Y	Y	Y	Y	Y	Y	Y	100

1. Was the sample frame appropriate to address the target population? 2. Were study participants sampled in an appropriate way? 3. Was the sample size adequate? 4. Were the study subjects and the setting described in detail? 5. Was the data analysis conducted with sufficient coverage of the identified sample? 6. Were valid methods used for the identification of the condition? 7. Was the condition measured in a standard, reliable way for all participants? 8. Was there appropriate statistical analysis? 9. Was the response rate adequate, and if not, was the low response rate managed appropriately? Y=Yes; N=No; U=Unclear or NA Not applicable

A

Study ID	Cases	Total	Seroprevalence	95% C.I.	Weight
**Overall IgG**					
Abdelmoniem 2021	23	203	11.3	[7.0; 15.7]	3.2%
Adetifa 2021	928	9922	9.4	[8.8; 9.9]	3.6%
Batchi-Bouyou 2021	81	754	10.7	[8.5; 13.0]	3.5%
Chibwana 2020	84	500	16.8	[13.5; 20.1]	3.3%
Etyang 2021	135	684	19.7	[16.8; 22.7]	3.4%
Fai 2021	240	999	24.0	[21.4; 26.7]	3.4%
Fwoloshi 2021	13	575	2.3	[1.0; 3.5]	3.5%
Goldblatt 2021	23	222	10.4	[6.4; 14.4]	3.2%
Halatoko 2020	8	955	0.8	[0.3; 1.4]	3.6%
Ifeorah 2021	48	113	42.5	[33.4; 51.6]	2.4%
Kagucia 2021	329	830	39.6	[36.3; 43.0]	3.3%
Kassem 2020	3	74	4.1	[0.0; 8.5]	3.2%
Katchunga 2021	242	684	35.4	[31.8; 39.0]	3.3%
Kempen 2020	3	99	3.0	[0.0; 6.4]	3.3%
Majiya 2020	47	185	25.4	[19.1; 31.7]	2.9%
Mostafa 2021	20	2282	0.9	[0.5; 1.3]	3.6%
Mukhtar 2021	36	455	7.9	[5.4; 10.4]	3.4%
Mukwege 2021	67	359	18.7	[14.6; 22.7]	3.2%
Mulenga 2021	80	2704	3.0	[2.3; 3.6]	3.6%
Musa 2021	241	1111	21.7	[19.3; 24.1]	3.4%
Nkuba 2021	167	1080	15.5	[13.3; 17.6]	3.5%
Nwosu 2021	302	971	31.1	[28.2; 34.0]	3.4%
Olayanju 2021	60	133	45.1	[36.7; 53.6]	2.5%
Rusakaniko 2021	29	635	4.6	[2.9; 6.2]	3.5%
Sebastião 2021	7	660	1.1	[0.3; 1.8]	3.5%
Shaw 2021	96	405	23.7	[19.6; 27.8]	3.2%
Shaweno 2021	22	684	3.2	[1.9; 4.5]	3.5%
Uyoga 2021	174	3098	5.6	[4.8; 6.4]	3.5%
Wiens 2021	411	1840	22.3	[20.4; 24.2]	3.5%
Zarmouh 2021	22	897	2.5	[1.4; 3.5]	3.5%
**Random effects model**		**34113**	**14.6**	**[12.2; 17.1]**	**100.0%**

Heterogeneity: I^2 = 99%, τ^2 = 0.0043, χ^2_{29} = 3321.89 (p = 0)

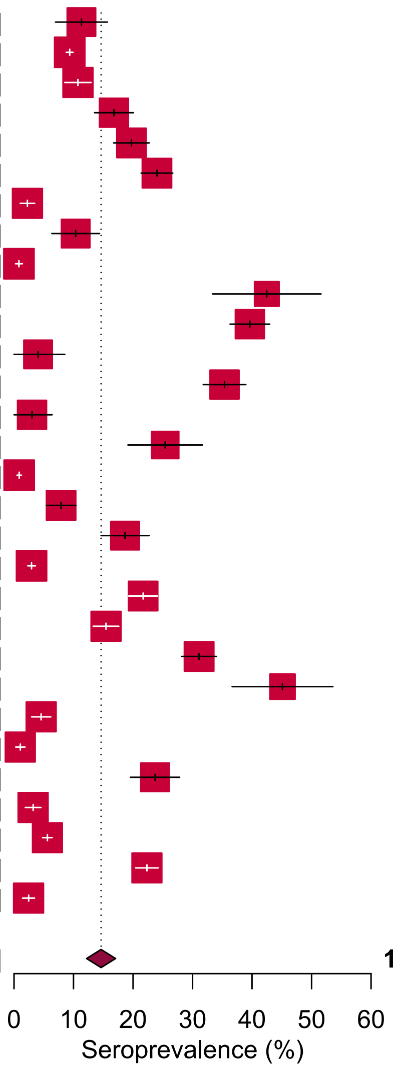

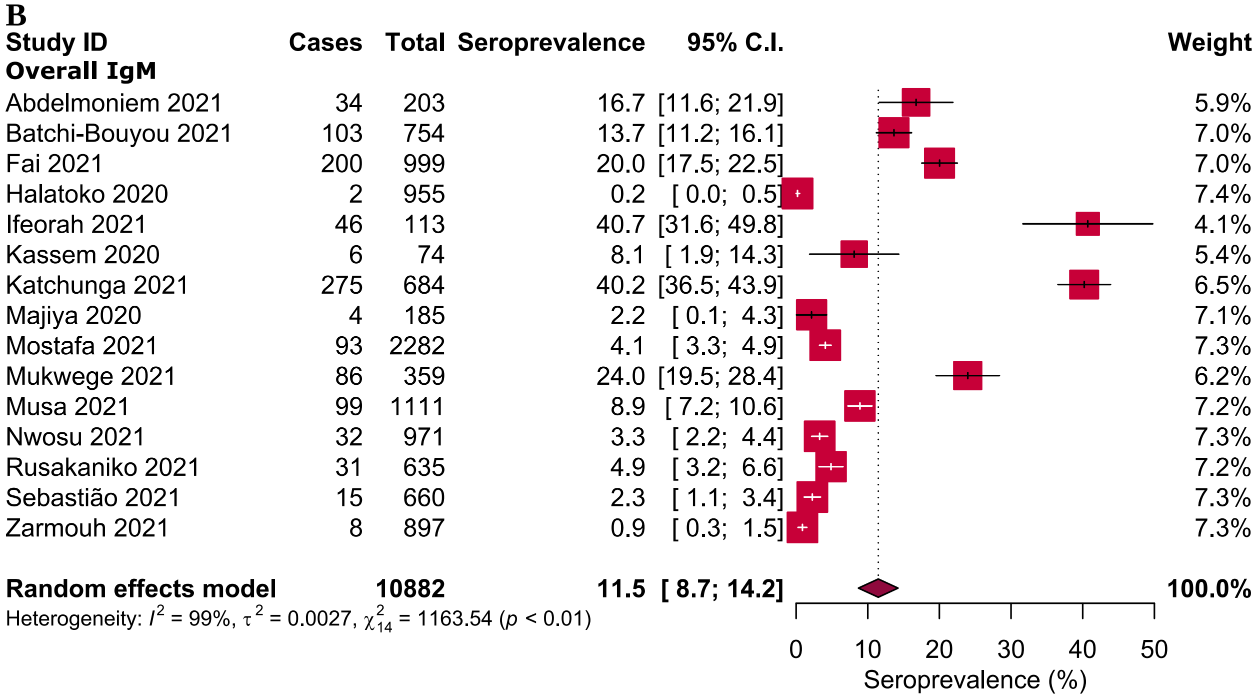
B
Study ID Cases Total Seroprevalence 95% C.I. Weight
Overall IgM
Abdelmoniem 2021 34 203 16.7 [11.6; 21.9] 5.9%
Batchi-Bouyou 2021 103 754 13.7 [11.2; 16.1] 7.0%
Fai 2021 200 999 20.0 [17.5; 22.5] 7.0%
Halatoko 2020 2 955 0.2 [0.0; 0.5] 7.4%
Ifeorah 2021 46 113 40.7 [31.6; 49.8] 4.1%
Kassem 2020 6 74 8.1 [1.9; 14.3] 5.4%
Katchunga 2021 275 684 40.2 [36.5; 43.9] 6.5%
Majiya 2020 4 185 2.2 [0.1; 4.3] 7.1%
Mostafa 2021 93 2282 4.1 [3.3; 4.9] 7.3%
Mukwege 2021 86 359 24.0 [19.5; 28.4] 6.2%
Musa 2021 99 1111 8.9 [7.2; 10.6] 7.2%
Nwosu 2021 32 971 3.3 [2.2; 4.4] 7.3%
Rusakaniko 2021 31 635 4.9 [3.2; 6.6] 7.2%
Sebastião 2021 15 660 2.3 [1.1; 3.4] 7.3%
Zarmouh 2021 8 897 0.9 [0.3; 1.5] 7.3%
Random effects model 10882 11.5 [8.7; 14.2] 100.0%
Heterogeneity: I^2 = 99%, τ^2 = 0.0027, χ^2_{14} = 1163.54 (p < 0.01)
0 10 20 30 40 50
Seroprevalence (%)

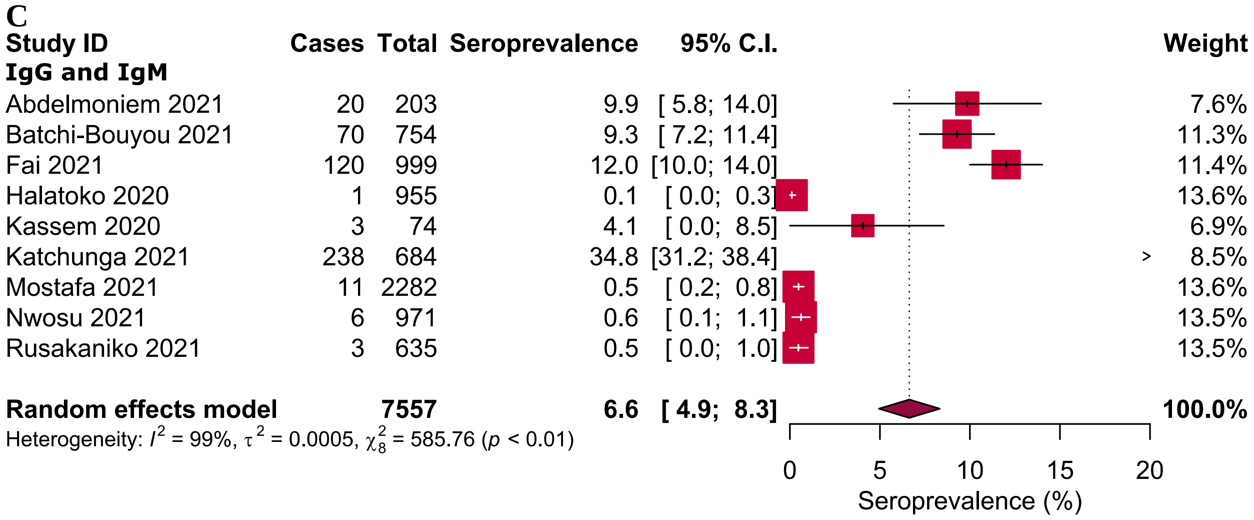
C
Study ID Cases Total Seroprevalence 95% C.I. Weight
IgG and IgM
Abdelmoniem 2021 20 203 9.9 [5.8; 14.0] 7.6%
Batchi-Bouyou 2021 70 754 9.3 [7.2; 11.4] 11.3%
Fai 2021 120 999 12.0 [10.0; 14.0] 11.4%
Halatoko 2020 1 955 0.1 [0.0; 0.3] 13.6%
Kassem 2020 3 74 4.1 [0.0; 8.5] 6.9%
Katchunga 2021 238 684 34.8 [31.2; 38.4] 8.5%
Mostafa 2021 11 2282 0.5 [0.2; 0.8] 13.6%
Nwosu 2021 6 971 0.6 [0.1; 1.1] 13.5%
Rusakaniko 2021 3 635 0.5 [0.0; 1.0] 13.5%
Random effects model 7557 6.6 [4.9; 8.3] 100.0%
Heterogeneity: I^2 = 99%, τ^2 = 0.0005, χ^2_{8} = 585.76 (p < 0.01)
0 5 10 15 20
Seroprevalence (%)

D

Rapid diagnostic test

Study ID	Cases	Total	Seroprevalence	95% C.I.	Weight
Abdella 2021	56	1856	3.0	[2.2; 3.8]	6.9%
Abdelmoniem 2021	37	203	18.2	[12.9; 23.5]	6.3%
Assefa 2021	83	1447	5.7	[4.5; 6.9]	6.8%
Batchi-Bouyou 2021	149	754	19.8	[16.9; 22.6]	6.7%
Fai 2021	320	999	32.0	[29.1; 34.9]	6.7%
Halatoko 2020	9	955	0.9	[0.3; 1.6]	6.9%
Kammon 2020	6	219	2.7	[0.6; 4.9]	6.8%
Kassem 2020	9	74	12.2	[4.7; 19.6]	5.8%
Katchunga 2021	279	684	40.8	[37.1; 44.5]	6.6%
Milleliri 2021	422	1687	25.0	[22.9; 27.1]	6.8%
Mostafa 2021	100	2282	4.4	[3.5; 5.2]	6.9%
Nega 2020	23	301	7.6	[4.6; 10.6]	6.7%
Nwosu 2021	328	971	33.8	[30.8; 36.8]	6.7%
Quashie 2021	252	1305	19.3	[17.2; 21.5]	6.8%
Rusakaniko 2021	57	635	9.0	[6.8; 11.2]	6.8%
**Random effects model**		**14372**	**15.5**	**[11.0; 20.1]**	**100.0%**

Heterogeneity: $I^2 = 99\%$, $\tau^2 = 0.0079$, $\chi^2_{14} = 1921.90$ ($p = 0$)

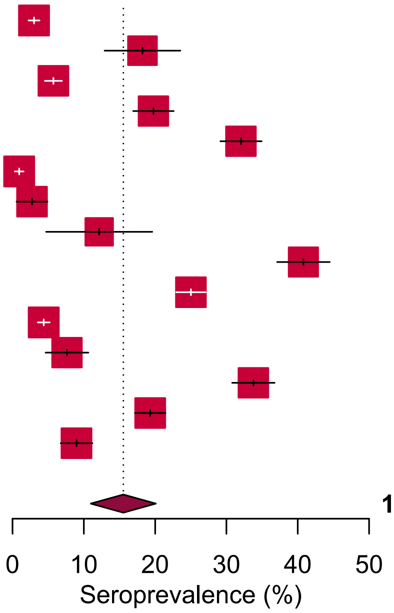

E

Enzyme-linked immunosorbent assay

Study ID	Cases	Total	Seroprevalence	95% C.I.	Weight
Adetifa 2021	928	9922	9.4	[8.8; 9.9]	10.6%
Chibwana 2020	84	500	16.8	[13.5; 20.1]	10.0%
Etyang 2021	135	684	19.7	[16.8; 22.7]	10.1%
Fwoloshi 2021	13	575	2.3	[1.0; 3.5]	10.5%
Goldblatt 2021	23	222	10.4	[6.4; 14.4]	9.7%
Kagucia 2021	329	830	39.6	[36.3; 43.0]	10.0%
Mulenga 2021	80	2704	3.0	[2.3; 3.6]	10.6%
Olayanju 2021	60	133	45.1	[36.7; 53.6]	7.5%
Uyoga 2021	174	3098	5.6	[4.8; 6.4]	10.6%
Wiens 2021	411	1840	22.3	[20.4; 24.2]	10.4%
**Random effects model**		**20508**	**16.5**	**[12.2; 20.8]**	**100.0%**

Heterogeneity: $I^2 = 99\%$, $\tau^2 = 0.0046$, $\chi^2_{9} = 1090.15$ ($p < 0.01$)

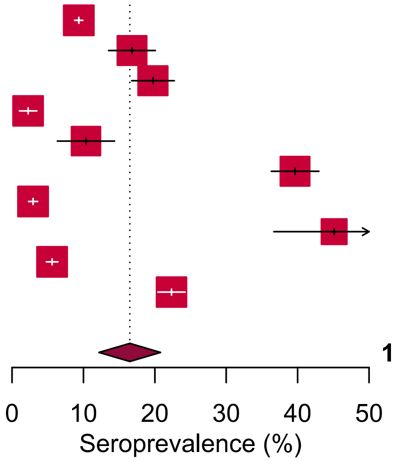

F

Chemiluminescent microparticle immunoassay

Study ID	Cases	Total	Seroprevalence	95% C.I.	Weight
Kempen 2020	3	99	3.0	[0.0; 6.4]	33.2%
Shaw 2021	96	405	23.7	[19.6; 27.8]	32.7%
Shaweno 2021	22	684	3.2	[1.9; 4.5]	34.1%
**Random effects model**		**1188**	**9.8**	**[0.0; 20.7]**	**100.0%**

Heterogeneity: $I^2 = 98\%$, $\tau^2 = 0.0089$, $\chi^2_{2} = 86.58$ ($p < 0.01$)

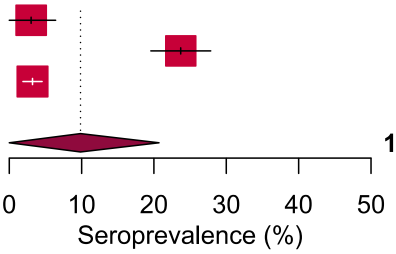

G

Study ID	Cases	Total	Seroprevalence	95% C.I.	Weight
**Clinical Laboratory Improvement Amendments**					
George 2021	1802	6477	27.8	[26.7; 28.9]	50.0%
Zarmouh 2021	30	897	3.3	[2.2; 4.5]	50.0%
**Random effects model**		**7374**	**15.6**	**[0.0; 39.6]**	**100.0%**

Heterogeneity: $I^2 = 100\%$, $\tau^2 = 0.0299$, $\chi^2_1 = 893.66$ ($p < 0.01$)

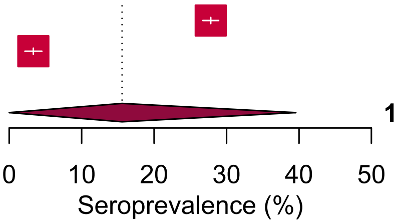

H

Study ID	Cases	Total	Seroprevalence	95% C.I.	Weight
**General population**					
Abdella 2021	56	1856	3.0	[2.2; 3.8]	7.8%
Batchi-Bouyou 2021	149	754	19.8	[16.9; 22.6]	7.6%
Kammon 2020	6	219	2.7	[0.6; 4.9]	7.7%
Kempen 2020	3	99	3.0	[0.0; 6.4]	7.5%
Mulenga 2021	80	2704	3.0	[2.3; 3.6]	7.8%
Nega 2020	23	301	7.6	[4.6; 10.6]	7.6%
Ngere 2021	384	1164	33.0	[30.3; 35.7]	7.6%
Nkuba 2021	167	1080	15.5	[13.3; 17.6]	7.7%
Nwosu 2021	328	971	33.8	[30.8; 36.8]	7.6%
Sebastião 2021	20	660	3.0	[1.7; 4.3]	7.8%
Shaweno 2021	22	684	3.2	[1.9; 4.5]	7.8%
Wiens 2021	411	1840	22.3	[20.4; 24.2]	7.7%
Zarmouh 2021	30	897	3.3	[2.2; 4.5]	7.8%
**Random effects model**		**13229**	**11.7**	**[7.4; 16.0]**	**100.0%**

Heterogeneity: $I^2 = 99\%$, $\tau^2 = 0.0060$, $\chi^2_{12} = 1372.65$ ($p < 0.01$)

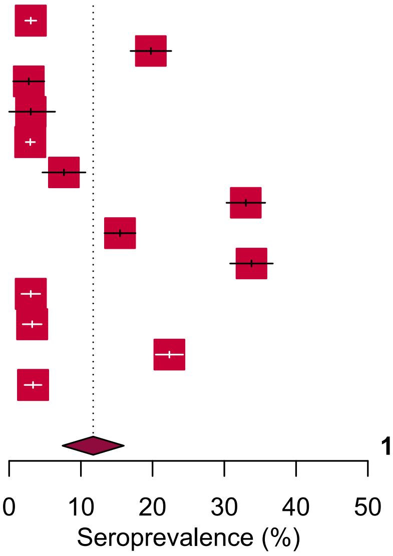

I

Study ID	Cases	Total	Seroprevalence	95% C.I.	Weight
**Healthcare workers**					
Abdelmoniem 2021	37	203	18.2	[12.9; 23.5]	8.8%
Chibwana 2020	84	500	16.8	[13.5; 20.1]	9.4%
Etyang 2021	135	684	19.7	[16.8; 22.7]	9.5%
Fwoloshi 2021	13	575	2.3	[1.0; 3.5]	9.8%
Goldblatt 2021	23	222	10.4	[6.4; 14.4]	9.2%
Kassem 2020	9	74	12.2	[4.7; 19.6]	8.0%
Mostafa 2021	100	2282	4.4	[3.5; 5.2]	9.8%
Mukhtar 2021	36	455	7.9	[5.4; 10.4]	9.6%
Mukwege 2021	148	359	41.2	[36.1; 46.3]	8.9%
Olayanju 2021	60	133	45.1	[36.7; 53.6]	7.6%
Rusakaniko 2021	57	635	9.0	[6.8; 11.2]	9.6%
**Random effects model**		**6122**	**16.3**	**[11.5; 21.2]**	**100.0%**

Heterogeneity: $I^2 = 98\%$, $\tau^2 = 0.0063$, $\chi^2_{10} = 474.06$ ($p < 0.01$)

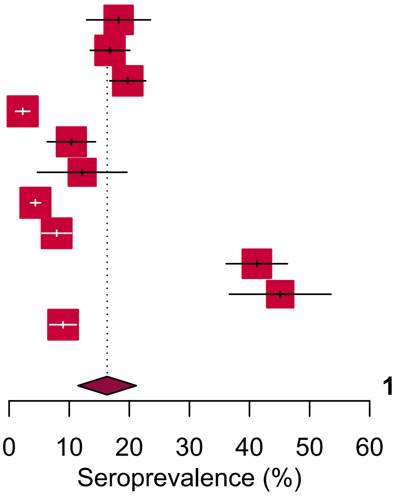

J

Study ID	Cases	Total	Seroprevalence	95% C.I.	Weight
**Blood donors**					
Adetifa 2021	928	9922	9.4	[8.8; 9.9]	50.3%
Uyoga 2021	174	3098	5.6	[4.8; 6.4]	49.7%
**Random effects model**		**13020**	**7.5**	**[3.8; 11.2]**	**100.0%**

Heterogeneity: $I^2 = 98\%$, $\tau^2 = 0.0007$, $\chi^2_1 = 54.42$ ($p < 0.01$)

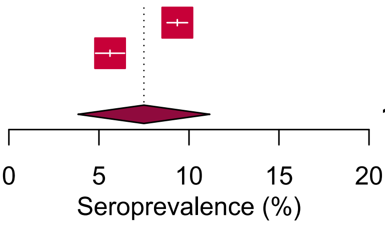

K

Study ID	Cases	Total	Seroprevalence	95% C.I.	Weight
**Pregnant women**					
Assefa 2021	83	1447	5.7	[4.5; 6.9]	100.0%

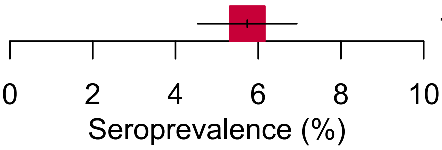

L

Study ID	Cases	Total	Seroprevalence	95% C.I.	Weight
**Community**					
Abdella 2021	56	1856	3.0	[2.2; 3.8]	7.2%
Batchi-Bouyou 2021	149	754	19.8	[16.9; 22.6]	7.1%
Kagucia 2021	329	830	39.6	[36.3; 43.0]	7.0%
Milleliri 2021	422	1687	25.0	[22.9; 27.1]	7.2%
Mulenga 2021	80	2704	3.0	[2.3; 3.6]	7.2%
Nega 2020	23	301	7.6	[4.6; 10.6]	7.1%
Ngere 2021	384	1164	33.0	[30.3; 35.7]	7.1%
Nkuba 2021	167	1080	15.5	[13.3; 17.6]	7.2%
Nwosu 2021	328	971	33.8	[30.8; 36.8]	7.1%
Sebastião 2021	20	660	3.0	[1.7; 4.3]	7.2%
Shaw 2021	96	405	23.7	[19.6; 27.8]	6.9%
Shaweno 2021	22	684	3.2	[1.9; 4.5]	7.2%
Wiens 2021	411	1840	22.3	[20.4; 24.2]	7.2%
Zarmouh 2021	30	897	3.3	[2.2; 4.5]	7.2%
**Random effects model**		**15833**	**16.7**	**[11.7; 21.8]**	**100.0%**

Heterogeneity: $I^2 = 99\%$, $\tau^2 = 0.0093$, $\chi^2_{13} = 2134.79$ ($p = 0$)

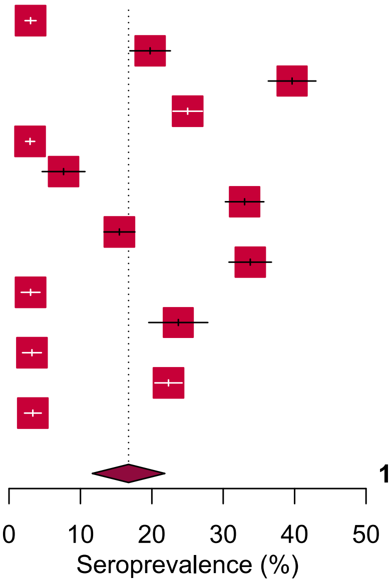

M

Study ID	Cases	Total	Seroprevalence	95% C.I.	Weight
**Hospital**					
Abdelmoniem 2021	37	203	18.2	[12.9; 23.5]	11.0%
Chibwana 2020	84	500	16.8	[13.5; 20.1]	11.4%
Etyang 2021	135	684	19.7	[16.8; 22.7]	11.5%
George 2021	1802	6477	27.8	[26.7; 28.9]	11.7%
Goldblatt 2021	23	222	10.4	[6.4; 14.4]	11.3%
Kassem 2020	9	74	12.2	[4.7; 19.6]	10.4%
Mukhtar 2021	36	455	7.9	[5.4; 10.4]	11.6%
Mukwege 2021	148	359	41.2	[36.1; 46.3]	11.1%
Olayanju 2021	60	133	45.1	[36.7; 53.6]	10.0%
**Random effects model**		**9107**	**21.9**	**[14.8; 29.0]**	**100.0%**

Heterogeneity: $I^2 = 98\%$, $\tau^2 = 0.0111$, $\chi^2_8 = 359.67$ ($p < 0.01$)

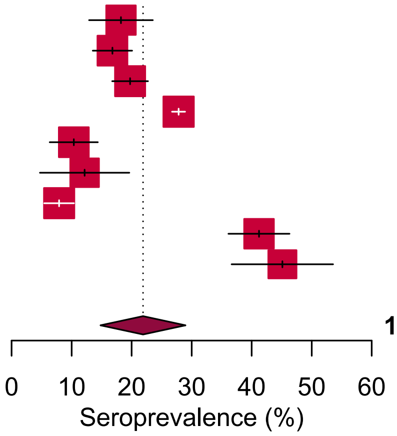

N

Study ID	Cases	Total	Seroprevalence	95% C.I.	Weight
**Healthcare facilities**					
Assefa 2021	83	1447	5.7	[4.5; 6.9]	17.0%
Fwoloshi 2021	13	575	2.3	[1.0; 3.5]	17.0%
Katchunga 2021	279	684	40.8	[37.1; 44.5]	16.0%
Kempen 2020	3	99	3.0	[0.0; 6.4]	16.1%
Mostafa 2021	100	2282	4.4	[3.5; 5.2]	17.1%
Rusakaniko 2021	57	635	9.0	[6.8; 11.2]	16.7%
**Random effects model**		**5722**	**10.6**	**[5.1; 16.1]**	**100.0%**

Heterogeneity: $I^2 = 99\%$, $\tau^2 = 0.0046$, $\chi^2_5 = 398.06$ ($p < 0.01$)

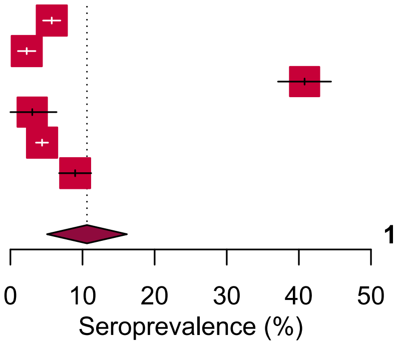

O

Study ID	Cases	Total	Seroprevalence	95% C.I.	Weight
**Blood transfusion centre**					
Adetifa 2021	928	9922	9.4	[8.8; 9.9]	50.3%
Uyoga 2021	174	3098	5.6	[4.8; 6.4]	49.7%
**Random effects model**		**13020**	**7.5**	**[3.8; 11.2]**	**100.0%**

Heterogeneity: $I^2 = 98\%$, $\tau^2 = 0.0007$, $\chi^2_1 = 54.42$ ($p < 0.01$)

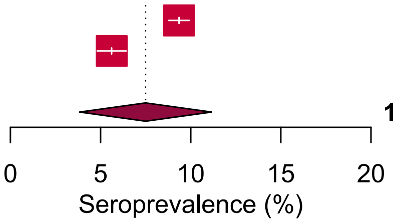

P

Study ID	Cases	Total	Seroprevalence	95% C.I.	Weight
**Northern Africa**					
Abdelmoniem 2021	37	203	18.2	[12.9; 23.5]	10.8%
Kammon 2020	6	219	2.7	[0.6; 4.9]	19.4%
Kassem 2020	9	74	12.2	[4.7; 19.6]	7.1%
Mostafa 2021	100	2282	4.4	[3.5; 5.2]	22.4%
Mukhtar 2021	36	455	7.9	[5.4; 10.4]	18.5%
Zarmouh 2021	30	897	3.3	[2.2; 4.5]	21.9%
**Random effects model**		**4130**	**6.5**	**[4.1; 8.9]**	**100.0%**

Heterogeneity: $I^2 = 88\%$, $\tau^2 = 0.0006$, $\chi^2_5 = 43.27$ ($p < 0.01$)

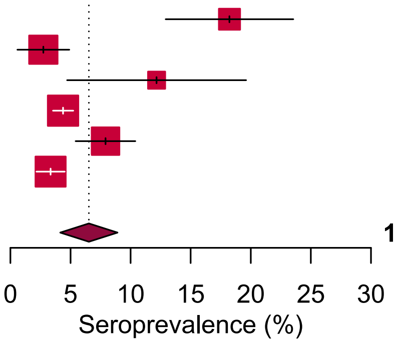

Q

Study ID	Cases	Total	Seroprevalence	95% C.I.	Weight
**Central Africa**					
Batchi-Bouyou 2021	149	754	19.8	[16.9; 22.6]	14.3%
Fai 2021	320	999	32.0	[29.1; 34.9]	14.3%
Katchunga 2021	279	684	40.8	[37.1; 44.5]	14.2%
Mukwege 2021	148	359	41.2	[36.1; 46.3]	14.1%
Nkuba 2021	167	1080	15.5	[13.3; 17.6]	14.4%
Nwosu 2021	328	971	33.8	[30.8; 36.8]	14.3%
Sebastião 2021	20	660	3.0	[1.7; 4.3]	14.4%
**Random effects model**		**5507**	**26.5**	**[14.6; 38.4]**	**100.0%**

Heterogeneity: I^2 = 99%, τ^2 = 0.0254, χ^2_6 = 907.48 (p < 0.01)

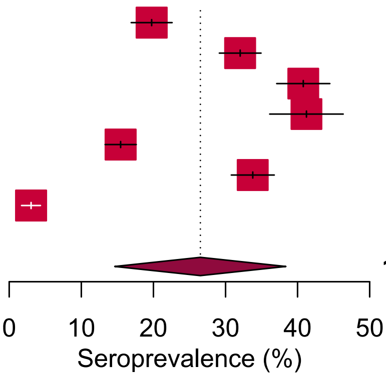

R

Study ID	Cases	Total	Seroprevalence	95% C.I.	Weight
**Eastern Africa**					
Abdella 2021	56	1856	3.0	[2.2; 3.8]	6.9%
Adetifa 2021	928	9922	9.4	[8.8; 9.9]	6.9%
Assefa 2021	83	1447	5.7	[4.5; 6.9]	6.8%
Chibwana 2020	84	500	16.8	[13.5; 20.1]	6.4%
Etyang 2021	135	684	19.7	[16.8; 22.7]	6.5%
Fwoloshi 2021	13	575	2.3	[1.0; 3.5]	6.8%
Kagucia 2021	329	830	39.6	[36.3; 43.0]	6.4%
Kempen 2020	3	99	3.0	[0.0; 6.4]	6.4%
Mulenga 2021	80	2704	3.0	[2.3; 3.6]	6.9%
Nega 2020	23	301	7.6	[4.6; 10.6]	6.5%
Ngere 2021	384	1164	33.0	[30.3; 35.7]	6.6%
Rusakaniko 2021	57	635	9.0	[6.8; 11.2]	6.7%
Shaweno 2021	22	684	3.2	[1.9; 4.5]	6.8%
Uyoga 2021	174	3098	5.6	[4.8; 6.4]	6.8%
Wiens 2021	411	1840	22.3	[20.4; 24.2]	6.7%
**Random effects model**		**26339**	**12.1**	**[8.8; 15.3]**	**100.0%**

Heterogeneity: I^2 = 99%, τ^2 = 0.0039, χ^2_{14} = 1505.68 (p < 0.01)

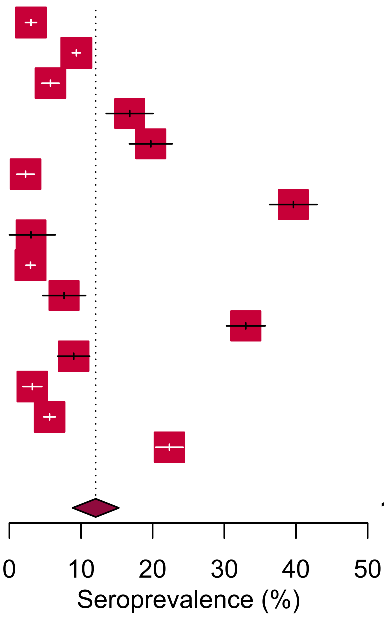

S

Study ID	Cases	Total	Seroprevalence	95% C.I.	Weight
**Western Africa**					
Halatoko 2020	9	955	0.9	[0.3; 1.6]	25.5%
Milleliri 2021	422	1687	25.0	[22.9; 27.1]	25.4%
Olayanju 2021	60	133	45.1	[36.7; 53.6]	23.7%
Quashie 2021	252	1305	19.3	[17.2; 21.5]	25.4%
**Random effects model**		**4080**	**22.2**	**[6.7; 37.6]**	**100.0%**

Heterogeneity: I^2 = 100%, τ^2 = 0.0243, χ^2_3 = 779.35 (p < 0.01)

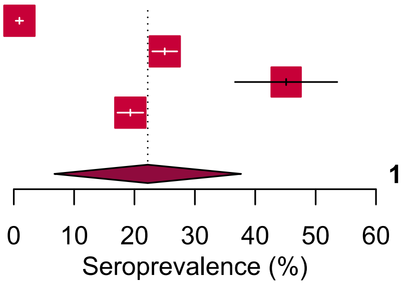

T

Study ID	Cases	Total	Seroprevalence	95% C.I.	Weight
**Southern Africa**					
George 2021	1802	6477	27.8	[26.7; 28.9]	34.4%
Goldblatt 2021	23	222	10.4	[6.4; 14.4]	32.8%
Shaw 2021	96	405	23.7	[19.6; 27.8]	32.7%
**Random effects model**		**7104**	**20.7**	**[10.4; 31.1]**	**100.0%**

Heterogeneity: $I^2 = 97\%$, $\tau^2 = 0.0081$, $\chi^2_2 = 69.64$ ($p < 0.01$)

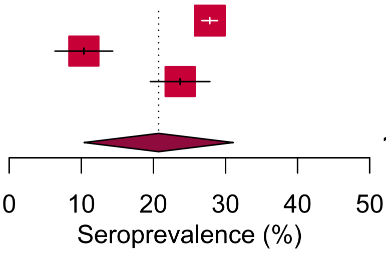

U

Study ID	Cases	Total	Seroprevalence	95% C.I.	Weight
**Angola**					
Sebastião 2021	20	660	3.0	[1.7; 4.3]	100.0%

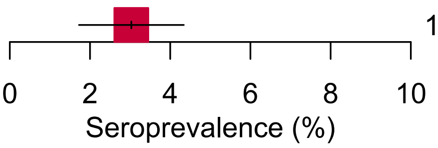

V

Study ID	Cases	Total	Seroprevalence	95% C.I.	Weight
**Cameroon**					
Fai 2021	320	999	32.0	[29.1; 34.9]	51.4%
Nwosu 2021	328	971	33.8	[30.8; 36.8]	48.6%
**Random effects model**		**1970**	**32.9**	**[30.8; 35.0]**	**100.0%**

Heterogeneity: $I^2 = 0\%$, $\tau^2 = 0$, $\chi^2_1 = 0.68$ ($p = 0.41$)

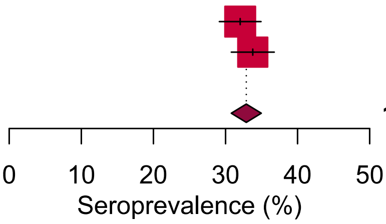

W

Study ID	Cases	Total	Seroprevalence	95% C.I.	Weight
**Republic of the Congo**					
Batchi-Bouyou 2021	149	754	19.8	[16.9; 22.6]	100.0%

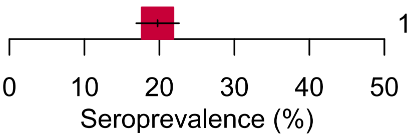

X

Study ID	Cases	Total	Seroprevalence	95% C.I.	Weight
**Democratic Republic of the Congo**					
Katchunga 2021	279	684	40.8	[37.1; 44.5]	33.4%
Mukwege 2021	148	359	41.2	[36.1; 46.3]	33.0%
Nkuba 2021	167	1080	15.5	[13.3; 17.6]	33.6%
**Random effects model**		**2123**	**32.4**	**[13.1; 51.7]**	**100.0%**

Heterogeneity: $I^2 = 99\%$, $\tau^2 = 0.0287$, $\chi^2_2 = 183.96$ ($p < 0.01$)

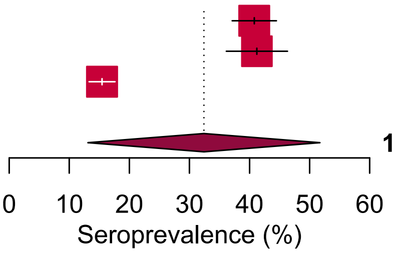

Y

Study ID	Cases	Total	Seroprevalence	95% C.I.	Weight
**Egypt**					
Abdelmoniem 2021	37	203	18.2	[12.9; 23.5]	22.8%
Kassem 2020	9	74	12.2	[4.7; 19.6]	18.3%
Mostafa 2021	100	2282	4.4	[3.5; 5.2]	30.4%
Mukhtar 2021	36	455	7.9	[5.4; 10.4]	28.5%
**Random effects model**		**3014**	**10.0**	**[5.0; 15.0]**	**100.0%**

Heterogeneity: $I^2 = 91\%$, $\tau^2 = 0.0021$, $\chi^2_3 = 34.67$ ($p < 0.01$)

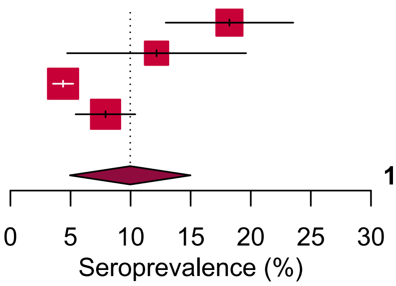

Z

Study ID	Cases	Total	Seroprevalence	95% C.I.	Weight
**Ethiopia**					
Abdella 2021	56	1856	3.0	[2.2; 3.8]	26.3%
Assefa 2021	83	1447	5.7	[4.5; 6.9]	24.2%
Kempen 2020	3	99	3.0	[0.0; 6.4]	12.2%
Nega 2020	23	301	7.6	[4.6; 10.6]	13.8%
Shaweno 2021	22	684	3.2	[1.9; 4.5]	23.5%
**Random effects model**		**4387**	**4.4**	**[2.8; 5.9]**	**100.0%**

Heterogeneity: $I^2 = 81\%$, $\tau^2 = 0.0002$, $\chi^2_4 = 21.15$ ($p < 0.01$)

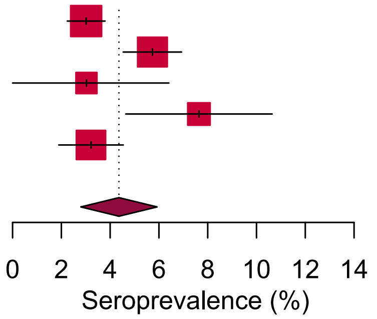

AA

Study ID	Cases	Total	Seroprevalence	95% C.I.	Weight
**Ghana**					
Quashie 2021	252	1305	19.3	[17.2; 21.5]	100.0%

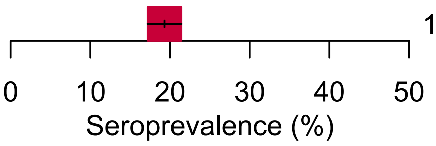

AB

Study ID	Cases	Total	Seroprevalence	95% C.I.	Weight
**Ivory Coast**					
Milleliri 2021	422	1687	25.0	[22.9; 27.1]	100.0%

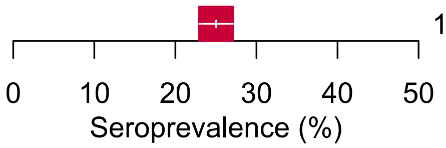

AC

Study ID	Cases	Total	Seroprevalence	95% C.I.	Weight
**Kenya**					
Adetifa 2021	928	9922	9.4	[8.8; 9.9]	20.4%
Etyang 2021	135	684	19.7	[16.8; 22.7]	19.8%
Kagucia 2021	329	830	39.6	[36.3; 43.0]	19.6%
Ngere 2021	384	1164	33.0	[30.3; 35.7]	19.9%
Uyoga 2021	174	3098	5.6	[4.8; 6.4]	20.3%
**Random effects model**		**15698**	**21.3**	**[13.6; 29.0]**	**100.0%**

Heterogeneity: $I^2 = 99\%$, $\tau^2 = 0.0075$, $\chi^2_4 = 738.84$ ($p < 0.01$)

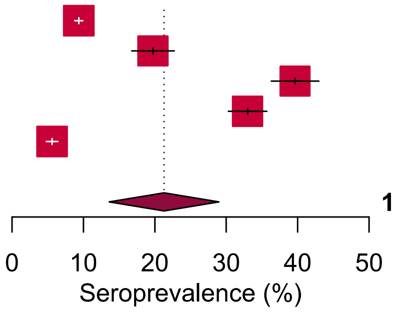

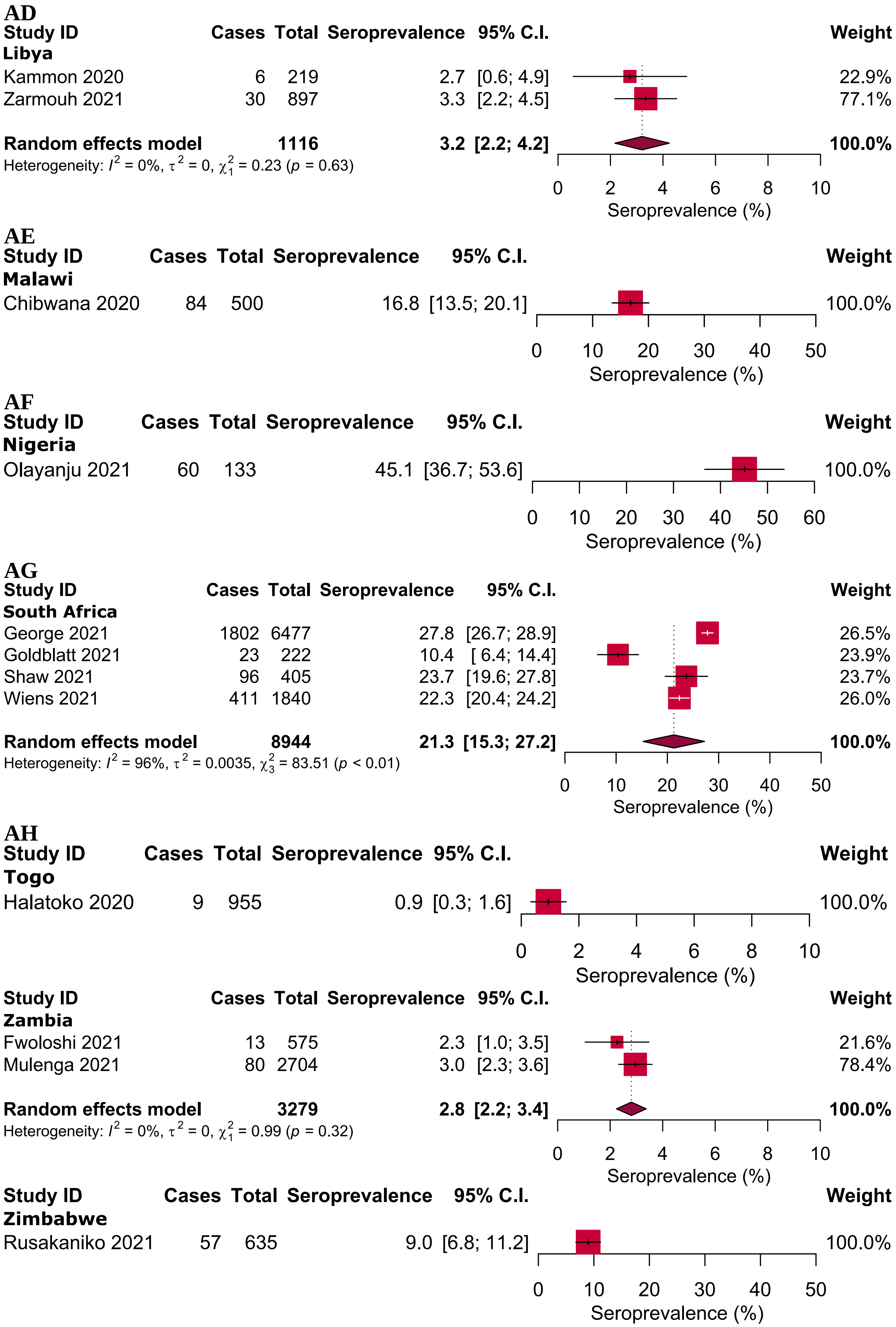
AD
Study ID Cases Total Seroprevalence 95% C.I. Weight
Libya
Kammon 2020 6 219 2.7 [0.6; 4.9] 22.9%
Zarmouh 2021 30 897 3.3 [2.2; 4.5] 77.1%
Random effects model 1116 3.2 [2.2; 4.2] 100.0%
Heterogeneity: I^2 = 0%, τ^2 = 0, χ^2_1 = 0.23 (p = 0.63)
0 2 4 6 8 10
Seroprevalence (%)
AE
Study ID Cases Total Seroprevalence 95% C.I. Weight
Malawi
Chibwana 2020 84 500 16.8 [13.5; 20.1] 100.0%
0 10 20 30 40 50
Seroprevalence (%)
AF
Study ID Cases Total Seroprevalence 95% C.I. Weight
Nigeria
Olayanju 2021 60 133 45.1 [36.7; 53.6] 100.0%
0 10 20 30 40 50 60
Seroprevalence (%)
AG
Study ID Cases Total Seroprevalence 95% C.I. Weight
South Africa
George 2021 1802 6477 27.8 [26.7; 28.9] 26.5%
Goldblatt 2021 23 222 10.4 [6.4; 14.4] 23.9%
Shaw 2021 96 405 23.7 [19.6; 27.8] 23.7%
Wiens 2021 411 1840 22.3 [20.4; 24.2] 26.0%
Random effects model 8944 21.3 [15.3; 27.2] 100.0%
Heterogeneity: I^2 = 96%, τ^2 = 0.0035, χ^2_3 = 83.51 (p < 0.01)
0 10 20 30 40 50
Seroprevalence (%)
AH
Study ID Cases Total Seroprevalence 95% C.I. Weight
Togo
Halatoko 2020 9 955 0.9 [0.3; 1.6] 100.0%
0 2 4 6 8 10
Seroprevalence (%)
Study ID Cases Total Seroprevalence 95% C.I. Weight
Zambia
Fwoloshi 2021 13 575 2.3 [1.0; 3.5] 21.6%
Mulenga 2021 80 2704 3.0 [2.3; 3.6] 78.4%
Random effects model 3279 2.8 [2.2; 3.4] 100.0%
Heterogeneity: I^2 = 0%, τ^2 = 0, χ^2_1 = 0.99 (p = 0.32)
0 2 4 6 8 10
Seroprevalence (%)
Study ID Cases Total Seroprevalence 95% C.I. Weight
Zimbabwe
Rusakaniko 2021 57 635 9.0 [6.8; 11.2] 100.0%
0 10 20 30 40 50
Seroprevalence (%)

Figure S1. Subgroup analyses representing the seroprevalence of SARS-CoV-2 antibodies in Africa based on antibody isotypes (A-C), antibody tests (D-G), target population (H-K), settings (L-O), regions (P-T) and countries (U-AJ).

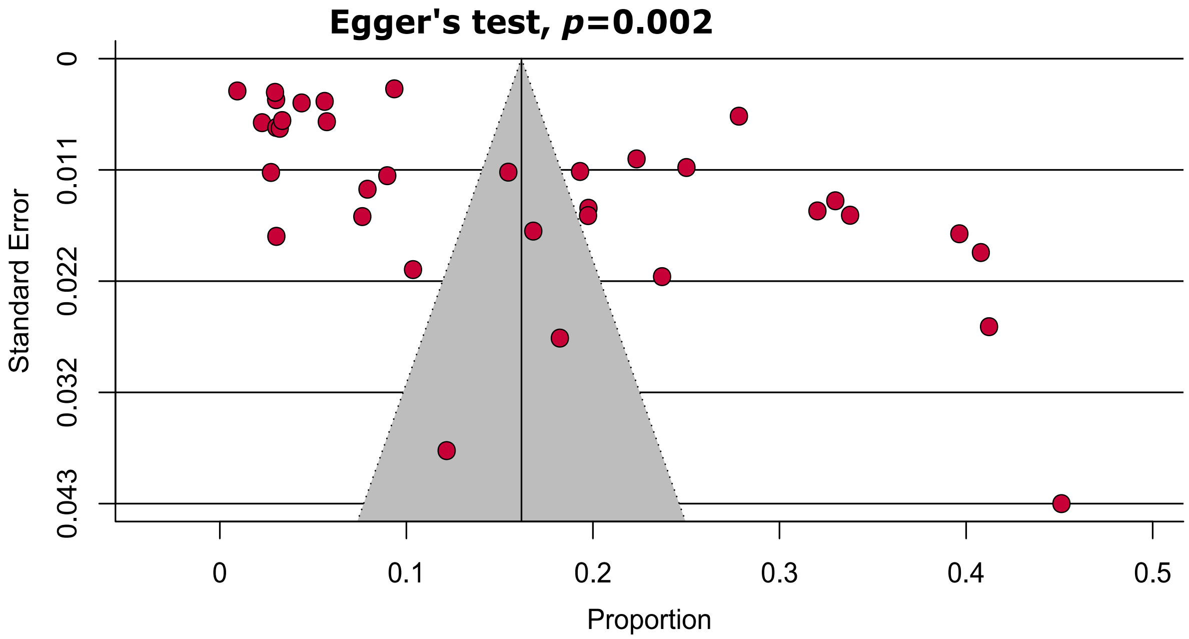

Figure S2. Funnel plot on overall seroprevalence of SARS-CoV-2 antibodies in Africa identified significant publication bias.

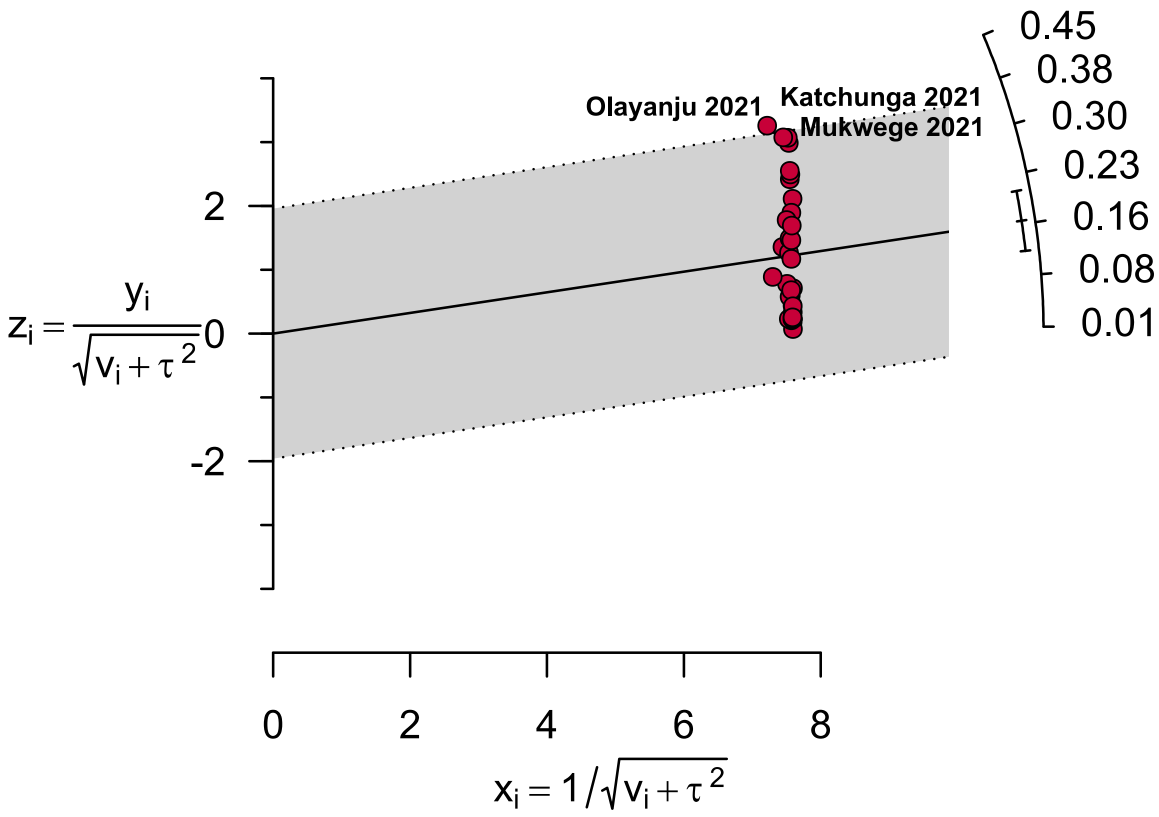

Figure S3. Galbraith plot on overall seroprevalence of SARS-CoV-2 antibodies in Africa identified three outlier studies.

A

Study ID	Cases	Total	Seroprevalence	95% C.I.	Weight
**Seroprevalence after excluding small studies**					
Abdella 2021	56	1856	3.0	[2.2; 3.8]	3.2%
Abdelmoniem 2021	37	203	18.2	[12.9; 23.5]	2.9%
Adetifa 2021	928	9922	9.4	[8.8; 9.9]	3.2%
Assefa 2021	83	1447	5.7	[4.5; 6.9]	3.2%
Batchi-Bouyou 2021	149	754	19.8	[16.9; 22.6]	3.1%
Chibwana 2020	84	500	16.8	[13.5; 20.1]	3.1%
Etyang 2021	135	684	19.7	[16.8; 22.7]	3.1%
Fai 2021	320	999	32.0	[29.1; 34.9]	3.1%
Fwoloshi 2021	13	575	2.3	[1.0; 3.5]	3.2%
George 2021	1802	6477	27.8	[26.7; 28.9]	3.2%
Goldblatt 2021	23	222	10.4	[6.4; 14.4]	3.0%
Halatoko 2020	9	955	0.9	[0.3; 1.6]	3.2%
Kagucia 2021	329	830	39.6	[36.3; 43.0]	3.1%
Kammon 2020	6	219	2.7	[0.6; 4.9]	3.1%
Katchunga 2021	279	684	40.8	[37.1; 44.5]	3.1%
Milleliri 2021	422	1687	25.0	[22.9; 27.1]	3.2%
Mostafa 2021	100	2282	4.4	[3.5; 5.2]	3.2%
Mukhtar 2021	36	455	7.9	[5.4; 10.4]	3.1%
Mukwege 2021	148	359	41.2	[36.1; 46.3]	2.9%
Mulenga 2021	80	2704	3.0	[2.3; 3.6]	3.2%
Nega 2020	23	301	7.6	[4.6; 10.6]	3.1%
Ngere 2021	384	1164	33.0	[30.3; 35.7]	3.1%
Nkuba 2021	167	1080	15.5	[13.3; 17.6]	3.1%
Nwosu 2021	328	971	33.8	[30.8; 36.8]	3.1%
Quashie 2021	252	1305	19.3	[17.2; 21.5]	3.1%
Rusakaniko 2021	57	635	9.0	[6.8; 11.2]	3.1%
Sebastião 2021	20	660	3.0	[1.7; 4.3]	3.2%
Shaw 2021	96	405	23.7	[19.6; 27.8]	3.0%
Shaweno 2021	22	684	3.2	[1.9; 4.5]	3.2%
Uyoga 2021	174	3098	5.6	[4.8; 6.4]	3.2%
Wiens 2021	411	1840	22.3	[20.4; 24.2]	3.2%
Zarmouh 2021	30	897	3.3	[2.2; 4.5]	3.2%
**Random effects model**		**46854**	**15.8**	**[12.8; 18.8]**	**100.0%**

Heterogeneity: $I^2 = 99\%$, $\tau^2 = 0.0074$, $\chi^2_{31} = 5121.56$ ($p = 0$)

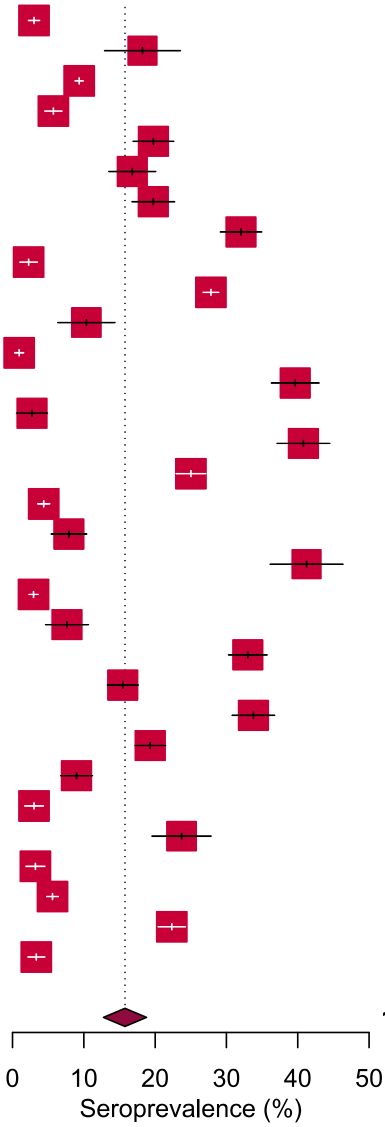

B

Study ID	Cases	Total	Seroprevalence	95% C.I.	Weight
**Seroprevalence after excluding low-quality studies**					
Abdella 2021	56	1856	3.0	[2.2; 3.8]	3.4%
Abdelmoniem 2021	37	203	18.2	[12.9; 23.5]	3.1%
Adetifa 2021	928	9922	9.4	[8.8; 9.9]	3.4%
Assefa 2021	83	1447	5.7	[4.5; 6.9]	3.4%
Batchi-Bouyou 2021	149	754	19.8	[16.9; 22.6]	3.3%
Chibwana 2020	84	500	16.8	[13.5; 20.1]	3.3%
Etyang 2021	135	684	19.7	[16.8; 22.7]	3.3%
Fai 2021	320	999	32.0	[29.1; 34.9]	3.3%
Fwoloshi 2021	13	575	2.3	[1.0; 3.5]	3.4%
George 2021	1802	6477	27.8	[26.7; 28.9]	3.4%
Goldblatt 2021	23	222	10.4	[6.4; 14.4]	3.2%
Halatoko 2020	9	955	0.9	[0.3; 1.6]	3.4%
Kagucia 2021	329	830	39.6	[36.3; 43.0]	3.3%
Kassem 2020	9	74	12.2	[4.7; 19.6]	2.9%
Katchunga 2021	279	684	40.8	[37.1; 44.5]	3.3%
Milleliri 2021	422	1687	25.0	[22.9; 27.1]	3.4%
Mostafa 2021	100	2282	4.4	[3.5; 5.2]	3.4%
Mukhtar 2021	36	455	7.9	[5.4; 10.4]	3.4%
Mukwege 2021	148	359	41.2	[36.1; 46.3]	3.1%
Mulenga 2021	80	2704	3.0	[2.3; 3.6]	3.4%
Ngere 2021	384	1164	33.0	[30.3; 35.7]	3.3%
Nkuba 2021	167	1080	15.5	[13.3; 17.6]	3.4%
Nwosu 2021	328	971	33.8	[30.8; 36.8]	3.3%
Rusakaniko 2021	57	635	9.0	[6.8; 11.2]	3.4%
Sebastião 2021	20	660	3.0	[1.7; 4.3]	3.4%
Shaw 2021	96	405	23.7	[19.6; 27.8]	3.2%
Shaweno 2021	22	684	3.2	[1.9; 4.5]	3.4%
Uyoga 2021	174	3098	5.6	[4.8; 6.4]	3.4%
Wiens 2021	411	1840	22.3	[20.4; 24.2]	3.4%
Zarmouh 2021	30	897	3.3	[2.2; 4.5]	3.4%
**Random effects model**		**45103**	**16.3**	**[13.1; 19.4]**	**100.0%**

Heterogeneity: $I^2 = 99\%$, $\tau^2 = 0.0075$, $\chi^2_{29} = 4987.01$ ($p = 0$)

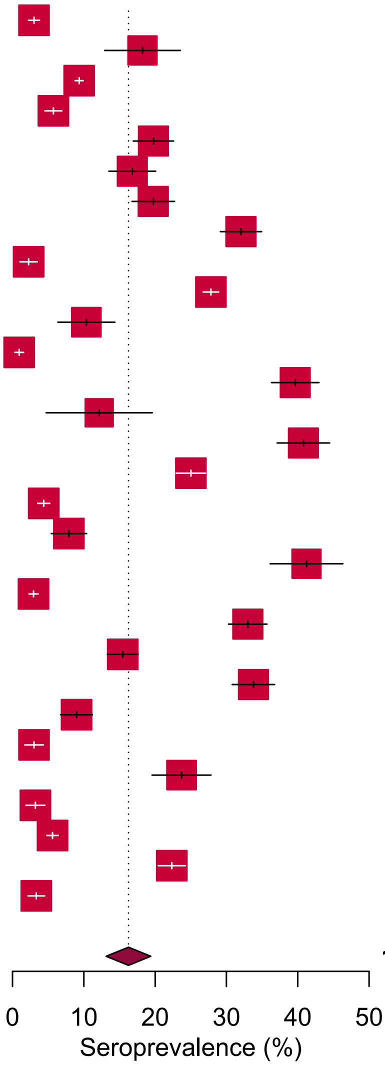

C

Study ID	Cases	Total	Seroprevalence	95% C.I.	Weight
**Seroprevalence after excluding outlier studies**					
Abdella 2021	56	1856	3.0	[2.2; 3.8]	3.2%
Abdelmoniem 2021	37	203	18.2	[12.9; 23.5]	2.9%
Adetifa 2021	928	9922	9.4	[8.8; 9.9]	3.2%
Assefa 2021	83	1447	5.7	[4.5; 6.9]	3.2%
Batchi-Bouyou 2021	149	754	19.8	[16.9; 22.6]	3.1%
Chibwana 2020	84	500	16.8	[13.5; 20.1]	3.1%
Etyang 2021	135	684	19.7	[16.8; 22.7]	3.1%
Fai 2021	320	999	32.0	[29.1; 34.9]	3.1%
Fwoloshi 2021	13	575	2.3	[1.0; 3.5]	3.2%
George 2021	1802	6477	27.8	[26.7; 28.9]	3.2%
Goldblatt 2021	23	222	10.4	[6.4; 14.4]	3.0%
Halatoko 2020	9	955	0.9	[0.3; 1.6]	3.2%
Kagucia 2021	329	830	39.6	[36.3; 43.0]	3.1%
Kammon 2020	6	219	2.7	[0.6; 4.9]	3.2%
Kassem 2020	9	74	12.2	[4.7; 19.6]	2.6%
Kempen 2020	3	99	3.0	[0.0; 6.4]	3.1%
Milleliri 2021	422	1687	25.0	[22.9; 27.1]	3.2%
Mostafa 2021	100	2282	4.4	[3.5; 5.2]	3.2%
Mukhtar 2021	36	455	7.9	[5.4; 10.4]	3.1%
Mulenga 2021	80	2704	3.0	[2.3; 3.6]	3.2%
Nega 2020	23	301	7.6	[4.6; 10.6]	3.1%
Ngere 2021	384	1164	33.0	[30.3; 35.7]	3.1%
Nkuba 2021	167	1080	15.5	[13.3; 17.6]	3.2%
Nwosu 2021	328	971	33.8	[30.8; 36.8]	3.1%
Quashie 2021	252	1305	19.3	[17.2; 21.5]	3.2%
Rusakaniko 2021	57	635	9.0	[6.8; 11.2]	3.2%
Sebastião 2021	20	660	3.0	[1.7; 4.3]	3.2%
Shaw 2021	96	405	23.7	[19.6; 27.8]	3.0%
Shaweno 2021	22	684	3.2	[1.9; 4.5]	3.2%
Uyoga 2021	174	3098	5.6	[4.8; 6.4]	3.2%
Wiens 2021	411	1840	22.3	[20.4; 24.2]	3.2%
Zarmouh 2021	30	897	3.3	[2.2; 4.5]	3.2%
**Random effects model**		**45984**	**13.8**	**[10.9; 16.6]**	**100.0%**

Heterogeneity: $I^2 = 99\%$, $\tau^2 = 0.0067$, $\chi^2_{31} = 4643.76$ ($p = 0$)

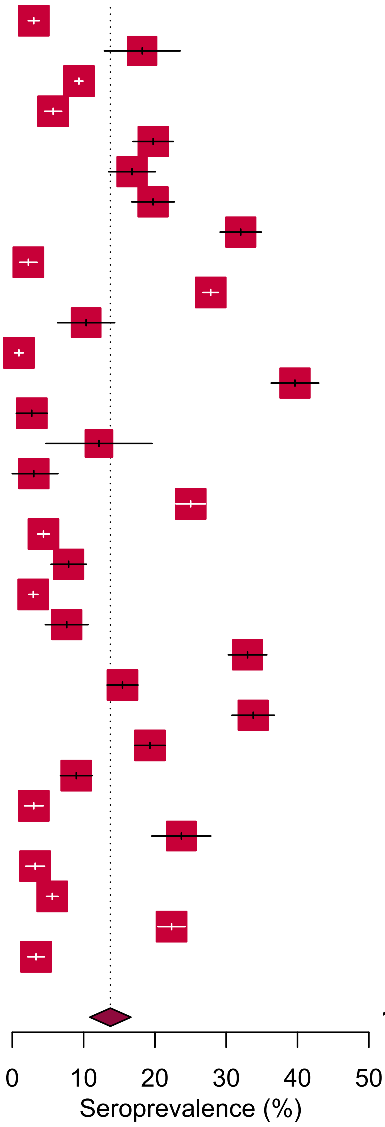

Figure S4. Sensitivity analyses by (A) excluding small studies (<200), (B) excluding low-quality studies and (C) excluding outlier studies.
